# Computerized clinical decision support systems for therapeutic drug monitoring and dosing: A decision-maker-researcher partnership systematic review

**DOI:** 10.1186/1748-5908-6-90

**Published:** 2011-08-03

**Authors:** Robby Nieuwlaat, Stuart J Connolly, Jean A Mackay, Lorraine Weise-Kelly, Tamara Navarro, Nancy L Wilczynski, R Brian Haynes

**Affiliations:** 1 Population Health Research Institute, McMaster University, Hamilton General Hospital campus, 237 Barton Street East, Hamilton, ON, Canada; 2 Department of Medicine, McMaster University, 1280 Main Street West, Hamilton, ON, Canada; 3 Hamilton Health Sciences, 1200 Main Street West, Hamilton, ON, Canada; 4 Health Information Research Unit, Department of Clinical Epidemiology and Biostatistics, McMaster University, 1280 Main Street West, Hamilton, ON, Canada

## Abstract

**Background:**

Some drugs have a narrow therapeutic range and require monitoring and dose adjustments to optimize their efficacy and safety. Computerized clinical decision support systems (CCDSSs) may improve the net benefit of these drugs. The objective of this review was to determine if CCDSSs improve processes of care or patient outcomes for therapeutic drug monitoring and dosing.

**Methods:**

We conducted a decision-maker-researcher partnership systematic review. Studies from our previous review were included, and new studies were sought until January 2010 in MEDLINE, EMBASE, Evidence-Based Medicine Reviews, and Inspec databases. Randomized controlled trials assessing the effect of a CCDSS on process of care or patient outcomes were selected by pairs of independent reviewers. A study was considered to have a positive effect (*i.e.*, CCDSS showed improvement) if at least 50% of the relevant study outcomes were statistically significantly positive.

**Results:**

Thirty-three randomized controlled trials were identified, assessing the effect of a CCDSS on management of vitamin K antagonists (14), insulin (6), theophylline/aminophylline (4), aminoglycosides (3), digoxin (2), lidocaine (1), or as part of a multifaceted approach (3). Cluster randomization was rarely used (18%) and CCDSSs were usually stand-alone systems (76%) primarily used by physicians (85%). Overall, 18 of 30 studies (60%) showed an improvement in the process of care and 4 of 19 (21%) an improvement in patient outcomes. All evaluable studies assessing insulin dosing for glycaemic control showed an improvement. In meta-analysis, CCDSSs for vitamin K antagonist dosing significantly improved time in therapeutic range.

**Conclusions:**

CCDSSs have potential for improving process of care for therapeutic drug monitoring and dosing, specifically insulin and vitamin K antagonist dosing. However, studies were small and generally of modest quality, and effects on patient outcomes were uncertain, with no convincing benefit in the largest studies. At present, no firm recommendation for specific systems can be given. More potent CCDSSs need to be developed and should be evaluated by independent researchers using cluster randomization and primarily assess patient outcomes related to drug efficacy and safety.

## Background

Healthcare policy makers and providers have already invested billions of dollars in information technology and systems to improve care effectiveness and efficiency, which will increase in the coming years. Optimization of the return on these investments requires that current best evidence be considered concerning the effects of information technology innovations on care processes and health outcomes.

Computerized clinical decision support systems (CCDSSs) may improve patient care by comparing individual patient features with a knowledge base to provide tailored clinical recommendations. One well-defined CCDSS clinical intervention area is therapeutic drug monitoring and dosing (TDMD). Certain drugs, such as warfarin or insulin, have variable effects depending on the plasma concentration in relation to individual patient-related factors. Managing such drugs is troublesome when they have a narrow therapeutic window--that is, a lower dose is ineffective and a somewhat higher dose is hazardous. To ensure an optimal net benefit, the drug effects need to be monitored with individually tailored dose adjustments accordingly. A CCDSS for TDMD could advise to monitor the drug effect within certain time intervals and advise specific dose adjustments based on this monitoring and the patient's characteristics.

Our 2005 review of 100 controlled trials of CCDSSs for all indications [1] included 24 studies assessing the effect of a CCDSS on TDMD: 13 for anticoagulants, four for theophylline, three for aminoglycosides, and four for other drugs. Practitioner performance improved in 15 (63%) of these studies and patient outcomes in 2 of 18 (11%) studies assessing this. Many CCDSS studies have been published since, with advancing information technology and, as we previously documented, increasingly strong research methods [1].

Our current systematic review, one of a series [2], aims to provide in-depth assessment of CCDSS effects on TDMD in randomized controlled trials (RCTs). In addition, the partnership of researchers and clinicians in the review process facilitated extraction and interpretation of details for practical implementation.

## Methods

The complete systematic review methods have been described in detail elsewhere [2]. Key and supplementary details for TDMD are provided here.

### Research question

Do CCDSSs improve process of care or patient outcomes for TDMD?

### Partnering with decision makers

To optimize the clinical relevance and applicability of results and conclusions for CCDSS implementation decisions, regional and local decision makers were involved throughout the entire review process. Overall direction for the review was provided by senior health policy makers for a large academic health sciences centre and regional health authority. Specific guidance for the area of TDMD was provided by a clinical service decision maker (SJC), chief of the regional cardiology program, who determined the clinical relevance of reported outcomes, helped integrating results across CCDSSs for different drugs, and provided clinical guidance for data analysis and the manuscript. The Health Information Research Unit research staff searched and selected studies, and extracted and synthesised data.

### Search strategy

**Figure 1:**
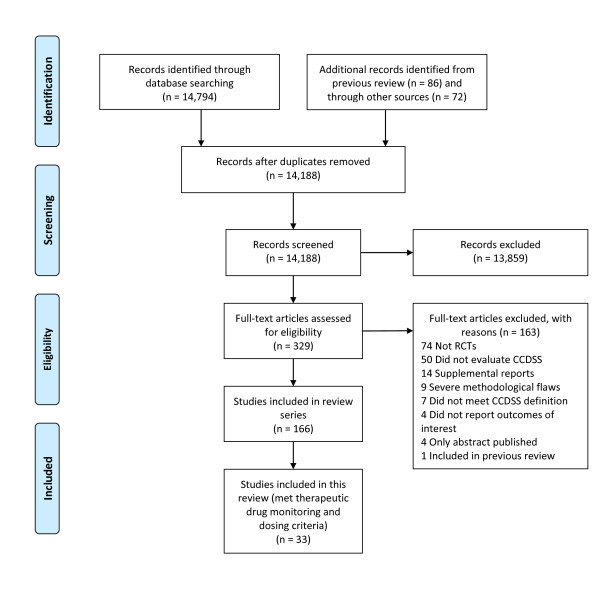
Flow diagram of included and excluded studies for the update 1 January 2004 to 6 January 2010 with specifics for therapeutic drug dosing and monitoring*. *Details provided in: Haynes RB *et al. *[2]. Two updating searches were performed, for 2004 to 2009 and to 6 January 2010 and the results of the search process are consolidated here.

We searched for RCTs with CCDSSs for all purposes until 6 January 2010 as cited in MEDLINE, EMBASE, Evidence-Based Medicine Reviews database, and the Inspec bibliographic database. We also reviewed reference lists of included studies and relevant review articles, and searched KT+ http://plus.mcmaster.ca/kt/ and EvidenceUpdates http://plus.mcmaster.ca/EvidenceUpdates/[3]. The flow diagram of included and excluded articles for the overall review is shown in Figure 1. Pairs of reviewers independently evaluated the eligibility of all identified studies. Cohen's kappa for reviewer agreement on study eligibility for all clinical areas together was κ = 0.93 (95% confidence interval (CI), 0.91 to 0.94). Disagreements were adjudicated by a third observer. Of the 33 included studies, reported in 36 publications [4-39], 16 overlapped [6-14,17,21,22,24,30,38,39] with the clinical area of 'acute care'; only their specific effect on TDMD will be reported here.

### Study selection

We included RCTs that assessed the effect of a CCDSS on process of care measures or patient outcomes, whereby the CCDSS provided dosing recommendations based on individual patient data and was handled by a healthcare professional. In our previous review [1], randomized and nonrandomized trials assessing the effect of a CCDSS on TDMD were identified until September 2004, and these studies were included in the current review if they were truly RCTs. An extended search until 6 January 2010 was performed to identify recent RCTs. CCDSSs that provided guidance on multiple management issues were included if the specific effect on TDMD could be isolated

### Data extraction

Pairs of reviewers independently extracted data. Disagreements were resolved by a third reviewer or by consensus. We attempted to contact primary authors via email to confirm accuracy of the extracted data and to provide missing data, and 25 of 33 (76%) replied. Researchers and clinical decision makers identified study variables relevant for each CCDSS intervention before evaluating intervention effects.

### Assessment of study quality

All RCTs were scored for methodological quality on a 10-point scale, which is an extension of the Jadad scale [1] and includes 5 potential sources of bias (see Additional file 1, Table S1). Total scores range from 0 (lowest study quality) to 10.

### Assessment of CCDSS intervention effects

CCDSS efficacy was assessed separately for process of care and patient outcomes based on variables relevant to the CCDSS intervention as judged by the researchers and clinical decision makers. A process of care outcome represents quality of care, such as the number of glucose measurements in the recommended therapeutic range. A patient outcome is directly measured patient's health, such as the number of symptomatic hypoglycaemic episodes. A CCDSS was considered effective when significantly (*p *< 0.05) improving the pre-specified primary endpoint. If no primary outcome was specified, then we based this determination on the endpoint used for study power calculation, or failing that, ≥50% of multiple pre-specified endpoints. When no endpoint was clearly pre-specified, we considered a CCDSS effective if it improved ≥50% of all reported outcomes. If the study compared more than one intervention with control, it was considered effective if any of the CCDSS study arms showed a benefit. These criteria are more specific than in our 2005 review [1], and the effect assignment was adjusted for some of the studies from that review.

### Data synthesis and analysis

CCDSS effects were analyzed with the study as the unit of analysis. If study designs and settings were considered comparable, data reported in ≥2 studies were pooled for meta-analysis to assess the average effect size. Where studies did not report data in a suitable form for pooling, authors were contacted for additional information, and appropriate data were estimated [40] with advice from a statistician. Data were combined as risk ratios for dichotomous data (Mantel-Haenszel method) or mean differences for continuous data (inverse variance method) using a random-effects model in Review Manager [41]. We interpreted a two-sided *p *< 0.05 as statistically significant. A sensitivity analysis was conducted to assess the possibility of biased results in studies with a mismatch between the unit of allocation (*e.g.*, clinicians) and the unit of analysis (*e.g.*, individual patients without adjustment for clustering). Success rates comparing studies with matched and mismatched analyses were compared using chi-square for comparisons. No differences in reported success were found for either process of care outcomes (Pearson X^2 ^= 1.12, 2*p *= 0.29) or patient outcomes (Pearson X^2 ^= 1.35, 2*p *= 0.53). Accordingly, results have been reported without distinction for mismatch.

## Results

From the previous 2005 review, 23 RCTs [4-26] for TDMD were included in the current review. An additional 10 RCTs, reported in 13 publications [27-39], were identified since September 2004. Three other studies were initially included, but later excluded for confounding of the CCDSS effect [42,43] or a quasi-randomized design [44]. Twenty included studies contribute outcomes to this review as well as other CCDSS interventions in the series; two studies [21,31] to four reviews, two studies [5,34] to three reviews, and 16 studies [6-14,17,22,24,30,32,38,39] to two reviews; but we focused here on relevant outcomes for therapeutic drug monitoring and dosing.

Summary of trial quality is reported in Additional file 1, Table S1; system characteristics in Additional file 2, Table S2; study characteristics in Additional file 3, Table S3; outcome data in Additional file 4, Table S4 and Table 1, and other CCDSS-related outcomes in Additional file 5, Table S5.

**Table 1:** Results for CCDSS trials of therapeutic drug monitoring and dosing^a^

Study	Methods score^b^	Indication	No. of centres/providers/patients	Process of care outcomes	CCDSS effect^c^	Patient outcomes	CCDSS effect^c^
**Vitamin K antagonist Dosing**							

Poller, 2008 [35-37]	5	1 of 2 CCDSSs (DAWN-AC or PARMA) provided dosing for warfarin/acenocoumarol/phenprocoumon in outpatients with AF, DVT or PE, mechanical heart valves, or other indications.	32/69/13,219*	Time INR in range (clinic-determined).	**+**	Adjudicated clinical events.	**0**
Claes, 2005 [27,28]	6	CCDSS (DAWN-AC) provided dosing for warfarin/acenocoumarol/phenprocoumon in outpatients with AF, DVT or PE, mechanical heart valves, or other indications.	66*/96/834	Duration of INR values within 0.5 or 0.75 INR-units of target range (2.5 or 3.5 depending on indication).	**0**	Thromboembolic complications and hemorrhagic events.	**0**
Mitra, 2005 [29]	5	CCDSS (DAWN-AC) provided dosing for warfarin in hospitalised rehabilitation patients,	1/.../30*	Time in therapeutic INR range (2.0 to 3.0) and number of blood draws during hospitalization.	**+**	Incident deep vein thrombosis or pulmonary embolism during hospitalization and length of hospital stay.	**..**.
Manotti, 2001 [26]	4	CCDSS (PARMA) provided dosing for warfarin/acenocoumarol in outpatients with VTE, non-ischemic heart disease, heart-valve prosthesis, or other indications.	5/.../1,251*	Time long term therapy group spent in therapeutic INR range (2.0 to 3.0 or 3.0 to 4.5) and proportion of starting treatment group reaching a stable condition (three consecutive INRs within therapeutic range, 2.0 to 3.0, at least one week from each other].	**+**	...	**..**.
Fitzmauric, 2000 [25]	6	CCDSS provided warfarin dosing for outpatients with venous or arterial thromboembolic disorders.	12*/.../367	Proportion of patients achieving therapeutic INR target, and time in target INR range (target range varied by clinical indication for treatment: 2.0 to 3.0 or 3.0 to 4.5).	**0**	Deaths, serious adverse events, and patient satisfaction.	**0**
Ageno, 1998 [23]	6	CCDSS (DAWN-AC) provided dosing for warfarin maintenance in outpatients with mechanical heart valves.	1/.../101*	INR within therapeutic range, >5.0, or <2.0;% dose adjustments; number of INR tests; time within INR range 2.5 to 3.5; mean INR; test interval; proportion interventions manually overridden in CCDSS group.	**..**.	...	**..**.
Poller, 1998 [24]	3	CCDSS (DAWN-AC) provided dosing for warfarin initiation and maintenance in outpatients.	5/.../285*	Time in INR target range (2 to 3 or 2.5 to 3.5, or 3 to -0.5).	**+**	...	**..**.
Vadher, 1997 [22]	6	CCDSS provided dosing for warfarin initiation and maintenance in inpatients with venous or arterial thromboembolic disorders.	1/49/148*	Time to reach therapeutic range and stable dose, time to pseudoevent (INR ≤1.5 or ≥5 after therapeutic range is reached), and time within INR range 2 to 3.	**0**	Deaths, thrombotic events, and hemorrhagic events.	**..**.
Fitzmauric, 1996 [20]	4	CCDSS provided dosing for warfarin maintenance in outpatients with venous or arterial thromboembolic disorders.	2/.../49*	INR control.	**..**.	Deaths, thrombotic or hemorrhagic episodes, and patient satisfaction.	**..**.
Fihn, 1994 [19]	3	CCDSS scheduled follow-up visits for outpatients receiving warfarin at anticoagulation clinics.	5/.../849*	Ability to increase visit intervals and deviation of measured prothrombin times and INRs from target values.	**+**	Deaths, clinically important bleeding, and thromboembolic complications.	**0**
Poller, 1993 [18]	5	CCDSS provided dosing for warfarin therapy in outpatients with venous or arterial thromboembolic disorders.	1/.../186*	Proportion of visits spent in or out of target range and time between visits.	**0**	Death, major bleeding events, and other clinical events	**0**
White, 1991 [15]	6	CCDSS predicted steady-state warfarin dosing in outpatients on long-term warfarin therapy.	1/.../50*	Difference between achieved and target PT, patients with final PT within 2 seconds of target, and follow-up interval.	**0**	...	**..**.
Carter, 1987 [9]	2	CCDSS provided dosing for warfarin initiation in hospital inpatients.	1/.../54*	Time from administration of first warfarin dose to stabilization dosage in patients with stable PT ratio pre-discharge	**0**	...	**..**.
White, 1987 [10]	6	CCDSS (Warfcalc) provided dosing for warfarin therapy in patients hospitalised with DVT, cerebrovascular accident, transient ischemic attack, PE, or AF.	2/.../75*	Time to reach stable therapeutic dose or therapeutic PR, patients with PR above therapeutic range during hospital stay, predicted vs observed PR, and absolute PR error.	**+**	Length of hospital stay and in-hospital bleeding complications.	**+**

**Aminophylline and Theophylline Dosing**							
Tierney, 2005 [31]	9	CCDSS generated care suggestions for physicians and pharmacists managing asthma and chronic obstructive pulmonary disease in adults in primary care.	4/266*/706	Proportion of care suggestions to change theophylline dose adhered to by physicians and pharmacists; medication compliance; and patient satisfaction with physicians and pharmacists.	**0**	Short-form 36 (physical function, role physical, pain, general health, vitality, social function, role emotional, mental health), asthma-related and chronic respiratory disease-related quality of life, emergency department visits, and hospitalizations.	**0**
Casner, 1993 [17]	3	CCDSS predicted theophylline infusion rates for inpatients with asthma or chronic obstructive pulmonary disease.	1/.../47*	Mean serum theophylline levels, absolute and mean difference between final and target (15 mg/L) theophylline levels, patients with subtherapeutic (<10 mg/L) final theophylline levels, and patients with toxic (>20 mg/L) final theophylline levels.	**0**	Theophylline-associated toxicity (nausea, vomiting, tremor, tachycardia, and seizures), length of hospital stay, treatment duration.	**0**
Gonzalez, 1989 [12]	5	CCDSS estimated aminophylline loading and maintenance dosing for patients in the emergency department.	.../.../67*	Mean theophylline level.	**+**	Discharge from emergency department within 8 hours, adverse effects in emergency department, and peak flow rate.	**0**
Hurley, 1986 [8]	8	CCDSS provided dosing for theophylline in inpatients with acute air-flow obstruction.	1/.../96*	Patients with theophylline levels above or below therapeutic range (10 to 20 μg/mL) on days 1 and 2 or trough theophylline levels in therapeutic range during oral therapy, mean serum theophylline levels, mean 1st serum level and trough levels during oral therapy.	**0**	In first 3 days: peak expiratory flow rate, air flow obstruction symptoms (severe breathlessness, wheeziness, night wheeze, or cough during hospitalization), side effects (severe palpitations, nausea, tremulousness, agitation, blurred vision, or diarrhoea during hospitalization), and deaths.	**0**

**Insulin Dosing and Glucose Glycaemic Regulation**							

Cavalcanti 2009 [39]	8	CCDSS (computer assisted insulin protocol, [CAIP]) recommended insulin dosing and glucose monitoring to achieve glucose control in patients in intensive care units.	5/60/168*	Number of blood glucose measurements and proportion of time blood glucose controlled (60 to 140 mg/dL).	**+**	Blood glucose levels in ICU and rates of hypoglycaemia.	**+/-**
Saager, 2008 [38]	6	CCDSS (EndoTool Glucose Management System) recommended insulin dosing and glucose assessment frequency for diabetic patients in cardiothoracic intensive care units.	1/.../40*	Proportion of blood glucose measures in range and time in range in operating rooms or intensive care units.	**+**	Blood glucose levels and time to reach blood glucose level <150 mg/dL in operating rooms or intensive care units.	+
Albisser, 2007 [33]	8	CCDSS predicted glycaemia and risk for hypoglycaemia in insulin-dependent patients in primary care.	.../2/22*	Mean daily insulin dose.	**+**	Hypoglycaemia episodes.	+
Rood, 2005 [30]	8	CCDSS recommended timing for glucose measurements and administration of insulin in critically ill patients.	1/104/484*	Proportion of time that glucose measurements were early or late, proportion of time that glucose levels were within target range (4.0 to 7.0 mmol/L), adherence to guideline for timing of glucose measurement, and proportion of samples taken on time.	**+**	**..**.	...
Ryff-de Léche, 1992 [16]	3	CCDSS (Camit S1) analyzed and summarized blood glucose data for Insulin dosing in outpatients with diabetes.	1/.../38*	Proportion of blood glucose levels in low range (<4.0 mmol/L), at <2.9 mmol/L level, and in target range (4.0 to 10.0 mmol/L).	**..**.	Change in haemoglobin A1c levels.	**..**.
McDonald, 1976 [5]	2	CCDSS generated recommendations for repeat laboratory tests to detect potential medication-related events and treatment changes in adults attending a diabetes clinic.	1/.../226*	Provider adherence to recommendations to change therapy or order tests for monitoring drug effects.	**+**	...	**..**.

**Aminoglycoside Dosing**							

Burton, 1991 [14]	6	CCDSS provided aminoglycoside dosing for inpatients with clinical infections.	1*/.../147	Proportion, of patients with peak aminoglycoside level >4 mg/L or trough levels ≥2 mg/L.	**0**	Deaths, cures, therapy response, treatment failure, indeterminate therapy response, nephrotoxicity, length of hospital stay overall and after start of antibiotics, and length of aminoglycoside therapy.	**0**
Begg, 1989 [11]	4	CCDSS provided individualised aminoglycoside dosing for inpatients receiving gentamicin or tobramycin.	.../.../50*	Number of patients achieving either or both peak (6 to 10 mg/L) and trough (1 to 2 mg/L) aminoglycoside levels.	**+**	Deaths and change in creatinine clearance during therapy.	**0**
Hickling, 1989 [13]	3	CCDSS provided dosing and dose intervals aminoglycoside in critically ill patients.	1/.../32*	Proportion of patients outside of therapeutic range (6 to 10 mg/L for peak and <2 mg/L for trough) or with peak plasma levels >6 mg/L., and mean peak and trough plasma aminoglycoside levels.	**+**	Increase in creatinine clearance during recovery.	**0**

**Digoxin Dosing/Monitoring**							

White, 1984 [7]	4	CCDSS (Health Evaluation through Logical Processing [HELP]) identified concerns (drug interactions or signs of potential digoxin intoxication) in inpatients taking digoxin.	1/.../396*	Physician compliance with alerts.	**+**	...	...
Peck, 1973 [4]	6	CCDSS provided a digoxin dosing scheme for outpatients with congestive heart failure.	1/4/42*	Errors for prediction of serum digoxin level.	**+**	Digoxin toxicity and congestive heart failure index.	**0**

**Lidocaine Dosing**							

Rodman, 1984 [6]	6	CCDSS recommended lidocaine dosing for patients in intensive or coronary care units.	1/.../20*	Plasma lidocaine levels in therapeutic range (1.5 to 5.0 μg/mL).	**+**	Toxic response requiring lidocaine discontinuation or dosage reduction.	**0**

**Miscellaneous**							

Matheny, 2008 [34]	8	CCDSS generated reminders for routine laboratory testing in primary care patients taking specified medications.	20*/303/1,922	Physician compliance with reminders.	**0**	...	**..**.
Judge, 2006 [32]	8	CCDSS provided real-time alerts when ordered drugs posed potential risks, required monitoring, or needed action to prevent adverse events in a long-term care setting.	1*/27/445	Physician compliance with alerts.	**0**	...	**..**.
Overhage, 1997 [21]	8	CCDSS determined corollary orders for 87 target orders and displayed these on-line to physicians using the CPOE. CCDSS identified corollary orders to prevent errors of omission for any of 87 target tests and treatments in hospital inpatients.	1*/92/2,181	Compliance with corollary orders and pharmacists interventions with physicians for significant errors.	**+**	Hospital length of stay and maximum serum creatinine level during hospital stay.	**0**

Abbreviations: AF, atrial fibrillation; CCDSS, computerized clinical decision support system; CPOE, computerized order entry system; INR, international normalized ratio; IV, intravenous; N/A, not available; PE, pulmonary embolism; PR, prothrombin ratio; PT, prothrombin time; SE, systemic embolism; VTE, venous thromboembolism.

*Unit of allocation.

^a^Ellipses (...) indicate item was not assessed or is not evaluable for effect.

^b^Score range 0 to 10, 10, higher quality score.

^c^Outcomes are evaluated for effect as positive (+) or negative (-) for CCDSS, or no effect (0), based on the following hierarchy. An effect is defined as ≥50% of relevant outcomes showing a statistically significant difference (2*p *< 0.05):

1. If a single primary outcome is reported, *in which all components are applicable*, this is the only outcome evaluated.

2. If > 1 primary outcome is reported, the ≥50% rule applies and only the primary outcomes are evaluated.

3. If no primary outcomes are reported (or only some of the primary outcome components are relevant) but overall analyses are provided, the overall analyses are evaluated as primary outcomes. Subgroup analyses are not considered.

4. If no primary outcomes or overall analyses are reported, or only some components of the primary outcome are relevant for the application, any reported prespecified outcomes are evaluated.

5. If no clearly prespecified outcomes are reported, any available outcomes are considered.

6. If statistical comparisons are not reported, 'effect' is designated as not evaluated (...).

### Study quality

The quality score of studies generally improved over time, mainly due to better follow-up of patients (see Additional file 1, Table S1). However, no studies had a perfect score and concealed study group allocation before randomization and cluster randomization were infrequent.

### CCDSS and study characteristics

CCDSSs were generally stand-alone computer systems (25/33, 76%) [4,6,8-20,22-25,27-29,33,35-39] (Additional file 2, Table S2). Most were used by physicians for decision making, (28/33, 85%) [4-19,21,23,24,26-37], the rest by other health professionals. Recommendations were usually delivered at the time of care (27/31, 87%) [4-7,10-14,16-19,21-26,29-32,34-39] on a desktop or laptop computer (16/25, 64%) [4,10,15,16,18,21,23-26,30-34,39]. Pilot testing was done in 48% (13/27) [6,8,9,16,20,22,24-26,30,33,34,39], training was provided to users in 55% (17/31) [6,7,9,10,12,17,19,20,24,25,27,28,30,31,33-37,39], and the authors were the developers of the CCDSS in 59% (17/29) of studies [5-7,10,11,13,16,19,21,22,26,30-34,39].

Additional file 3, Table S3 shows the characteristics of the 33 included RCTs [4-39]. A total of 24,627 patients were included, including one study with 13,219 patients and only six other studies [19,21,26-28,31,34-37] with more than 500 patients. The number of clinics within studies varied from 1 to 66, with the majority being performed at a single centre (63%) [4-9,13-18,21-23,29,30,32,38], and most involved academic centres (73%) [4-7,9,10,12,14,15,18-24,26,29,31,32,34-39]. Financial support was provided by public funding in 16 studies [4,6,8,9,14,19,21,22,24,25,31,32,34-39], private funding in eight studies [8,12,13,16,19,27,28,35-37,39] (four had both), and 13 studies [5,7,10,11,15,17,18,20,23,26,29,30,33] did not report a funding source.

### CCDSS effectiveness

Table 1 summarizes the effectiveness of all CCDSSs on TDMD and Additional file 4, Table S4 provides extensive outcome details. Overall, 60% of studies (18/30) [4-7,10-13,19,21,24,26,29,30,33,35-39] showed an improvement for process of care, and 21% (4/19) for patient outcomes [10,33,38,39]. It has to be noted that in Cavalcanti *et al. *the CCDSS scored positive on three of four patient outcomes and was therefore positive, but the proportion of patients with hypoglycaemia was actually worse than control [39]. Of seven cluster RCTs [14,21,25,27,28,31,32,34] only one showed an effect on process of care [21], and none showed an effect on patient outcomes. Not all studies assessed both types of outcomes, and we could not determine an effect for either outcome in three [16,20,23] because data were insufficient or not directly compared for CCDSS and control.

### Vitamin K antagonist dosing

**Figure 2:**
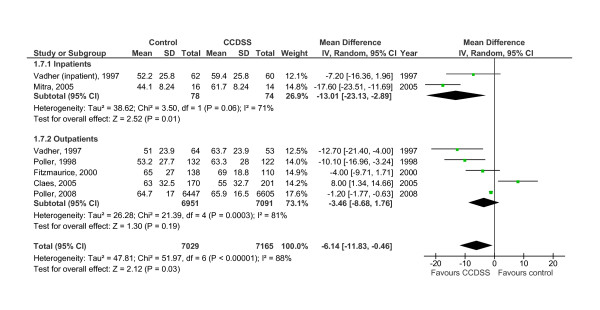
**Forest plot of comparison: Control versus CCDSS for proportion of time in INR range**.

Vitamin K antagonist (VKA) dosing RCTs (n = 14) [9,10,15,18-20,22-29,35-37] were generally of moderate quality (Table 1). Taking all VKA studies together, process of care was improved in 50% (6/12) [10,19,24,26,29,35-37] of studies with evaluable outcomes, and in meta-analysis the proportion of time in the therapeutic range for the blood International Normalized Ratio (INR) value was improved by CCDSSs (6.14%; 95% CI 0.46 to 11.83 increase; *p *= 0.03) (Figure 2). Patient outcomes were improved in 17% (1/6) of studies [10].

VKA initiation or inpatient therapy representing potentially unstable periods and assessed with a variety of outcomes in five RCTs [9,10,22,26,29] as shown in Additional file 4, Table S4, was improved in two studies (40%) [10,29]. When combining inpatient data from Vadher *et al. *[22] and Mitra *et al. *[29] in meta-analysis (Figure 2), CCDSS significantly improved the proportion of time in the therapeutic INR range for initiation therapy (13.01%; 95% CI, 2.89 to 23.13 increase; *p *= 0.01), but the effects were heterogeneous (I^2 ^= 71%) and the sample sizes small. White *et al. *[10] showed a shortened length of hospital stay with the CCDSS initiation therapy (see Additional file 4, Table S4).

**Figure 3:**
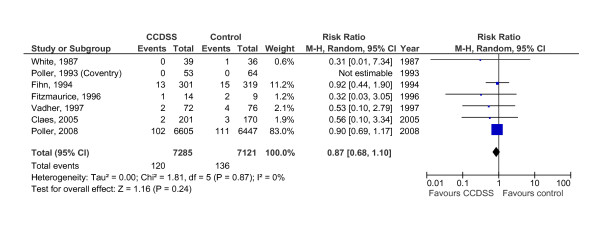
**Forest plot of comparison: CCDSS versus control for major bleeding**.

VKA maintenance therapy was assessed in 10 RCTs [15,18,19,22-28,35-37] by means of the proportion of time in the therapeutic INR range, INR return interval, or ability to achieve the target prothrombin time (PT) value, and five (50%) [19,22,24,26,35-37] showed an improvement (see Additional file 4, Table S4). When pooling five studies [22,24,25,27,28,35-37] with sufficient outpatient data on the proportion of time in the therapeutic INR range and its variability in meta-analysis (Figure 2), CCDSSs did not significantly improve anticoagulation quality compared with care as usual (3.46%; 95% CI, -1.76 to 8.68; *p *= 0.19), and the effects were heterogeneous (I^2 ^= 81%). Of note, the time in therapeutic INR range improved with CCDSS only 1.2% in the large study by Poller *et al. *and was worse than control in the study by *Claes et al. *Of 5 studies [18,25,27,28,35-37] assessing an effect on VKA maintenance therapy on patient outcomes, none found an improvement (see Additional file 4, Table S4). Combining major bleeding rates of 7 studies [10,18-20,22,27,35] in meta-analysis showed no significant lower risk with CCDSS (risk ratio of 0.87; 95% CI, 0.68 to 1.10; *p *= 0.24) compared with control (Figure 3).

### Theophylline/aminophylline dosing

Of four RCTs of theophylline or aminophylline dosing [8,12,17,31], only Gonzalez *et al. *[12] showed an improvement in process of care by means of a higher plasma theophylline level with the CCDSS in the first hours of intravenous aminophylline therapy (Table 1). Tierney *et al. *[31] showed no effect on primary care provider adherence to theophylline dosing recommendations, and both Casner *et al. *[17] and Hurley *et al. *[8] showed no effect on achieving therapeutic theophylline levels. No CCDSS significantly improved patient outcomes, including pulmonary function and drug toxicity.

### Insulin dosing/glycaemic regulation

Of six identified RCTs [5,16,30,33,38,39] assessing the effect of CCDSS on insulin dosing for glycaemic regulation, the four most recent studies [30,33,38,39] were of highest quality. All five evaluable studies [5,30,33,38,39] measuring process of care showed an improvement (Table 1). Among intensive care unit patients, Cavalcanti *et al. *[39] and Saager *et al. *[38] reported a higher proportion of time with glucose levels in the therapeutic target range. Albisser *et al. *[33] showed a decrease in the required insulin dose in primary care, Rood *et al. *[30] a better adherence to guideline recommendations for glucose measurement intervals and insulin dosing in critically ill patients, and McDonald [5] an increased adherence to a range of recommended laboratory tests and medication changes. Three studies assessed patient outcomes. Cavalcanti *et al. *[39] and Saager *et al. *[38] reported lower glucose levels but a higher rate of hypoglycaemia episodes with the CCDSS, and Saager *et al. *[38] found no change in admission duration. Albisser *et al. *[33] reported a decreased number of hypoglycaemia episodes, but no change in mean HbA_1c _levels.

### Aminoglycoside dosing

Three older RCTs [11,13,14] assessed CCDSSs' effect on dosing of aminoglycosides among inpatients with clinical infections. *Burton et al. *[14] showed no effect of the CCDSS on achieving both therapeutic peak and trough aminoglycoside levels, while *Begg et al. *[11] and the qualitatively poorer *Hickling et al. *[13] study found an improvement (see Additional file 4, Table S4). No significant effects were found on patient outcomes, encompassing mortality, therapy success, nephrotoxicity, and creatinine clearance (see Additional file 4, Table S4).

### Digoxin dosing

Two older RCTs [4,7] compared CCDSS-guided digoxin dosing with usual care. *White et al. *[7] showed an increase in recommended test ordering and digoxin dosing with the CCDSS in hospitalised patients. *Peck et al. *[4] showed improved digoxin serum level prediction among outpatients with heart failure, but showed no effect on patient outcomes.

### Lidocaine dosing

One RCT [6] tested a CCDSS for lidocaine dosing. Among patients admitted to the intensive care unit, the mean lidocaine plasma level achieved by the CCDSS was closer to the middle of the therapeutic target range than with usual care.

### Multiple treatment issues

Three cluster-RCTs [21,32,34] assessed the effect of a CCDSS on multiple drug therapy issues, including TDMD. *Matheny et al. *[34] showed no effect on overdue laboratory test ordering to assess therapeutic drug levels in primary care. In a long-term care setting, *Judge et al. *[32] reported a higher number of actions taken in relation to identified concerns with warfarin management, but no other TDMD related effects. *Overhage et al. *[21] showed an improvement in immediate compliance with on-line displayed corollary orders on a general medicine ward, including insulin, warfarin, digoxin and aminoglycosides, but these separate areas were not statistically tested. This CCDSS did not alter length of hospital stay or the maximum serum creatinine level.

### Costs and practical process related outcomes

*Ageno et al. *[23] reported that 4.9% of recommendations were overruled by the physician for vitamin K antagonist dosing, with rates of 10.9% for *Poller et al. *2008 [35-37] and <20% for *Manotti et al. *[26]. *Claes et al. *[27,28] described that the CoaguCheck, a point-of-care INR monitoring tool, scored higher for implementation preference than the CCDSS and regular performance feedback. *Rood et al. *[30] reported that a majority of practitioners were satisfied with the CCDSS, but no numbers were given. *Cavalcanti et al. *[39] found that nurses perceived the CCDSS to be equally complex and time consuming as conventional care, and 56% preferred adoption of the CCDSS.

Costs related to CCDSS use were reported in several studies, but few provided details on data collection and calculation methods. *Fitzmaurice et al. *[20] indicated that the initial costs of their warfarin CCDSS could be offset after 92 patient visits and *Fitzmaurice et al. *[25] reported that their warfarin CCDSS was associated with increased costs due to the initiation phase and increased INR testing. *Tierney et al. *[31] reported that the costs of a CCDSS for asthma and COPD management were significantly elevated when handled by physicians, *Burton et al. *[14] that there was a 4.09:1.00 benefit:cost ratio in favour of their CCDSS for aminoglycoside dosing, and *Overhage et al. *[21] that there was no effect on costs.

## Discussion

CCDSSs can improve the quality of insulin dosing for glycaemic control (improvement was based on at least 50% of the relevant study outcomes being statistically significantly positive), but longer term effects on patient outcomes are unknown. CCDSS improved the quality of vitamin K antagonist dosing as measured by time in therapeutic range, but effects were heterogeneous, RCTs were generally of modest size and quality, and a 13,000 patient study failed to show an effect on patient outcomes [35-37]. Current evidence is too inconclusive to recommend specific systems for TDMD. More potent CCDSS interventions need to be developed. Future trials should be performed by independent researchers, randomize non-specialised clinics, and primarily assess patient outcomes related to drug efficacy and safety.

### CCDSS effect on therapeutic drug monitoring and dosing

Overall, 60% of studies showed an improvement in process of care with the CCDSS, which is comparable to the 63% in the 2005 review [1]. Results were not consistent among studies, even when evaluating the same drug or the same CCDSS. This may relate to many factors, including variation in the study design, clinical setting, patient population, software specifications, and CCDSS workflow integration. Only 21% showed an improvement in patient outcomes, and although this is higher than the 11% in the 2005 review, most of these studies were still underpowered for this purpose [1]. A recently published review also summarized the evidence for the effect of CCDSSs on therapeutic drug dosing, but only reported studies until 2007, including three non-randomized controlled trials, and did not report the results of individual studies or drug classes but rather standardized effect measures [45]. Our report provides detailed information for RCT evidence per drug, while valuing the most relevant outcomes for decision-making.

The largest volume of RCT evidence was available for the effect of CCDSSs on vitamin K antagonist dosing, a group of oral anticoagulants widely used for thromboprophylaxis of which warfarin is the most common. The aim of a CCDSS for vitamin K antagonist dosing should be to establish a stable therapeutic INR in a timely manner, and to maintain INRs within the therapeutic range for the long term to minimize the risk for thromboembolism and bleeding. The improvement of 6% in time in therapeutic INR range among the studies in meta-analysis would theoretically mean a relative 6% reduced risk of stroke [46]. It could also mean the difference for a clinic of being above or below the threshold for a benefit of VKA over antiplatelet therapy [47]. However, most of these RCTs were small and of modest methodological quality, and the effect achieved statistical significance among inpatient but not outpatient studies. The CCDSS for vitamin K antagonist dosing with the most extensive RCT evidence, the DAWN AC program, improved process of care in three [24,29,35-37] of five [23,24,27-29,35-37] studies, but not patient outcomes in a large study designed to test this [35-37].

CCDSSs for insulin dosing generally kept glucose levels better in the target range, with an inconsistent lower risk for hypoglycaemia. Improving glycaemic control while minimizing hypoglycaemia may be essential to improve long-term patient outcomes, but this cannot be assumed, given recent trials of intensified diabetes care [48], and none of the CCDSS studies assessed meaningful longer term patient benefits.

Remaining drugs had less extensive RCT evidence. CCDSSs hardly improved dosing and safety for theophylline, a drug used prophylactically and acutely for respiratory diseases. Aminoglycosides, a group of antibiotics, digoxin, a cardiac glycoside, and lidocaine, an antiarrhythmic drug, had some indication that a CCDSS can improve dosing, but RCTs were of moderate quality and no new evidence has been reported in the past 20 years. Three studies assessing CCDSS effects on appropriate blood test ordering for potentially toxic drugs did not consistently show an improvement. This might indicate that a CCDSS for TDMD might not improve care by reminding healthcare providers of test ordering, but primarily by assistance for dose adjustment.

### Methodological aspects of CCDSSs and studies

Full intervention details were often incomplete, but are needed to understand how the CCDSS affects the clinical decision making process in order to advance the development and integration of effective systems. For example, we know that most CCDSSs were stand-alone systems, but to minimize the CCDSS burden on clinical workflow a shift from stand-alone systems requiring separate data entry to system integration with electronic medical records could be useful. Further, most CCDSSs were primarily handled by physicians, while a trained nurse handling the CCDSS with back up from a physician might further improve workflow in a cost-effective manner.

CCDSSs were often compared with specialists. This makes it harder to show an effect and it also limits the extrapolation of results because many patients taking vitamin K antagonists or insulin are being managed by non-specialised physicians, who will probably benefit most from a CCDSS. It will be essential to develop inexpensive and user-friendly systems to make CCDSSs attractive for non-specialists.

Although all studies were RCTs, many studies were of modest quality for remaining potential sources of bias. Few studies randomized clinical sites, rather than patients, which is an accepted method to minimize cross-over effects. The implementation of a CCDSS impacts care on an organizational level, and randomizing patients within the same clinic raises the concern of contaminating the control group with management effects from the CCDSS group, which could conceal a true effect. In contrast, finding an improvement while not using cluster randomization could indicate that there is surely a CCDSS effect. In our review, cluster RCTs less often showed an effect than patient randomized RCTs. However, considering that the latter were mostly single centre studies, this raises the concern that the local researcher might promote it to be a success, undermining the external validity of the positive result.

Another key methodological issue is the choice of outcome measures. A large variety of process measures were obtained, making it hard to compare or pool results of individual studies. TDMD should primarily be evaluated for rapidly achieving and adequately maintaining therapeutic serum levels of the monitored blood value. For example, one can report the mean maintenance dose for a vitamin K antagonist, but this provides little information because the main aim of the CCDSS should be to keep the INR within the therapeutic range, whichever dose this requires. CCDSS should also improve meaningful patient outcomes, and current research lacks this evidence. However, we do acknowledge that improving process measures that have previously improved outcomes is useful, because studies assessing patient outcomes typically require larger sample sizes and more funding.

### Limitations

Several studies reported incomplete data for evaluation of the CCDSS effects. Further, we included a variety of drugs and healthcare settings. These factors combined made it problematic to pool results, except for some VKA dosing studies. We chose not to convert overall results to general effect estimates for pooling of results, because this could provide spurious conclusions. We used 'vote counting' to assess the number of studies that were positive, whereby a study was considered to have a positive effect (*i.e.*, CCDSS showed improvement) if at least 50% of the relevant study outcomes were statistically significantly positive. This approach does not give insight in the magnitude of effects and may have underestimated the overall efficacy. On the other hand, there is a risk for publication bias of positive RCTs, which could cause overestimation of CCDSS efficacy. We reported limited details on CCDSS implementation in the final paragraph of the Results, but more extensive data are being collected from the authors and will be reported in a future publication on determinants of CCDSS success among all 166 RCTs. Some of the drugs are not as frequently used as at the time of the study, but their inclusion still adds to the general notion of CCDSS effects on TDMD. Finally, the knowledge basis for CCDSS recommendations should be well established, and weaker evidence underlying certain CCDSSs might have prevented strong adherence and thereby success.

### Clinical implications

Ideally, decision makers should consider a CCDSS that has shown a convincing effect on patient outcomes in more than one study, and value this against its burden on costs and workflow. Computer-assisted decision support for therapeutic drug monitoring and dosing is a promising area in development, especially for insulin and vitamin K antagonist dosing, but evaluations are unconvincing to date, and no specific system can be clearly recommended at this stage.

## Conclusions

CCDSSs have potential for improving process of care for TDMD, specifically insulin and vitamin K antagonist dosing. However, studies were generally small and of modest quality, effects on patient outcomes were uncertain, with no convincing benefit in the largest studies. At present, no firm recommendation for specific systems can be given. More potent CCDSSs need to be developed and should be evaluated by independent researchers using cluster randomization and primarily assess patient outcomes related to drug efficacy and safety.

## Competing interests

RBH, NLW, JAM, LWK, TN, RN, SJC received support through the Canadian Institutes of Health Research Synthesis Grant: Knowledge Translation KRS 91791 for the submitted work. RBH is acquainted with several CCDSS developers and researchers, including authors of papers included in this review.

## Authors' contributions

RBH was responsible for study conception and design; acquisition, analysis, and interpretation of data; drafting and critical revision of the manuscript; obtaining funding; and study supervision. He is the guarantor. RN acquired, analyzed, and interpreted data; drafted the manuscript; and provided statistical analysis. SJC analyzed and interpreted the data; and critically revised the manuscript. JAM acquired, analyzed, and interpreted data; drafted the manuscript; critically revised the manuscript; and provided statistical analysis as well as administrative, technical, or material support. LWK and TN acquired data and drafted the manuscript. NLW acquired, analyzed, and interpreted data; drafted the manuscript; provided administrative, technical, or material support; and provided study supervision. All authors have read and approved the final manuscript.

## Supplementary Material

Additional file 1 **Study methods scores for trials of therapeutic drug monitoring and dosing**. Methods scores for the included studies. Click here for file

Additional file 2 **CCDSS characteristics for trials of therapeutic drug monitoring and dosing**. CCDSS characteristics of the included studies. Click here for file

Additional file 3 **Study characteristics for trials of therapeutic drug monitoring and dosing**. Study characteristics of the included studies. Click here for file

Additional file 4 **Results for CCDSS trials of therapeutic drug monitoring and dosing**. Details results of the included studies. Click here for file

Additional file 5 **Costs and CCDSS process-related outcomes for trials of therapeutic drug monitoring and dosing**. Cost and CCDSS process-related outcomes for the included studies. Click here for file
