# Supplementary material for: Computerized clinical decision support systems for therapeutic drug monitoring and dosing: A decision-maker-researcher partnership systematic review
Source: Implement Sci. 2011 Aug 3;6:90. doi: 10.1186/1748-5908-6-90 (PMC3170236; doi:10.1186/1748-5908-6-90)
Supplement: Additional file 1 — Study methods scores for trials of therapeutic drug monitoring and dosing. Methods scores for the included studies. [file 1748-5908-6-90-S1.DOCX]

**Additional file 1, Table S1. Study methods scores for trials of therapeutic drug monitoring and dosing^a^**

| **Study** | **Allocation concealed^b^** | **Cluster randomization** | **Adjustment for baseline differences** | **Objective outcome** | **Adequate follow-up** | **Total score** |
| --- | --- | --- | --- | --- | --- | --- |
| Peck, 1973 [4] | 0 | 0 | 2 | 2 | 2 | 6 |
| McDonald, 1976 [5] | 0 | 0 | 0 | 2 | 0 | 2 |
| Rodman, 1984 [6] | 2 | 0 | 2 | 2 | 0 | 6 |
| White, 1984 [7] | 0 | 0 | 2 | 2 | 0 | 4 |
| Hurley, 1986 [8] | 2 | 0 | 2 | 2 | 2 | 8 |
| Carter, 1987 [9] | 0 | 0 | 0 | 2 | 0 | 2 |
| White, 1987 [10] | 0 | 0 | 2 | 2 | 2 | 6 |
| Begg, 1989 [11] | 0 | 0 | 2 | 2 | 0 | 4 |
| Gonzalez, 1989 [12] | 0 | 0 | 2 | 2 | 1 | 5 |
| Hickling, 1989 [13] | 0 | 0 | 0 | 2 | 1 | 3 |
| Burton, 1991 [14] | 0 | 2 | 2 | 2 | 0 | 6 |
| White, 1991 [15] | 0 | 0 | 2 | 2 | 2 | 6 |
| Ryff-de Lèche, 1992 [16] | 0 | 0 | 1 | 2 | 0 | 3 |
| Casner, 1993 [17] | 0 | 0 | 1 | 2 | 0 | 3 |
| Poller, 1993 [18] | 0 | 0 | 1 | 2 | 2 | 5 |
| Fihn, 1994 [19] | 0 | 0 | 1 | 2 | 0 | 3 |
| Fitzmaurice, 1996 [20] | 0 | 0 | 0 | 2 | 2 | 4 |
| Overhage, 1997 [21] | 2 | 2 | 2 | 2 | 0 | 8 |
| Vadher, 1997 [22] | 0 | 0 | 2 | 2 | 2 | 6 |
| Ageno, 1998 [23] | 0 | 0 | 2 | 2 | 2 | 6 |
| Poller, 1998 [24] | 0 | 0 | 0 | 2 | 1 | 3 |
| Fitzmaurice, 2000 [25] | 0 | 2 | 2 | 2 | 0 | 6 |
| Manotti, 2001 [26] | 0 | 0 | 2 | 2 | 0 | 4 |
| Claes, 2005 [27, 28] | 0 | 2 | 1 | 2 | 1 | 6 |
| Mitra, 2005 [29] | 0 | 0 | 1 | 2 | 2 | 5 |
| Rood, 2005 [30] | 2 | 0 | 2 | 2 | 2 | 8 |
| Tierney, 2005 [31] | 2 | 1 | 2 | 2 | 2 | 9 |
| Judge, 2006 [32] | 2 | 2 | 0 | 2 | 2 | 8 |
| Albisser, 2007 [33] | 2 | 0 | 2 | 2 | 2 | 8 |
| Matheny, 2008 [34] | 0 | 2 | 2 | 2 | 2 | 8 |
| Poller, 2008 [35-37] | 0 | 0 | 1 | 2 | 2 | 5 |
| Saager, 2008 [38] | 0 | 0 | 2 | 2 | 2 | 6 |
| Cavalcanti, 2009 [39] | 2 | 0 | 2 | 2 | 2 | 8 |

^a^Based on five individual items (score 2 = yes, 1 = partly, and 0 = no) and a summed total score (range 0 to 10). Because this review update included only randomized, controlled trials, the total score differs from that reported in the previous version of this review [1] the item evaluating study type (randomized, quasi-randomized, or concurrent controls) has been replaced by one that evaluates use of concealed allocation (concealed, unclear, not concealed).

^b^If allocation concealment is not readily apparent from the description provided in the published article, the primary author of the trial confirmed or indicated that allocation was concealed.
