# Supplementary material for: Computerized clinical decision support systems for therapeutic drug monitoring and dosing: A decision-maker-researcher partnership systematic review
Source: Implement Sci. 2011 Aug 3;6:90. doi: 10.1186/1748-5908-6-90 (PMC3170236; doi:10.1186/1748-5908-6-90)
Supplement: Additional file 2 — CCDSS characteristics for trials of therapeutic drug monitoring and dosing. CCDSS characteristics of the included studies. [file 1748-5908-6-90-S2.DOCX]

**Additional file 2, Table S2. CCDSS characteristics for trials of therapeutic drug monitoring and dosing^a^**

| **Study** | **Design** | | | **Interface description** | | | | | **Data entry source** | | | | | | **Methods for delivery of recommendations** | | | | | | | **CCDSS users** | | | | | | **Other characteristics** | | | | |
| --- | --- | --- | --- | --- | --- | --- | --- | --- | --- | --- | --- | --- | --- | --- | --- | --- | --- | --- | --- | --- | --- | --- | --- | --- | --- | --- | --- | --- | --- | --- | --- | --- |
|  | **Stand Alone** | **Integrated with EMR** | **Integrated with CPOE** | Graphic user interface | User must type | Drop down menus | Drag and drop | Other interface | **Automated through EMR** | **Project staff** | **Existing staff** | **Practitioner/decision-maker** | **Patient** | **Other data entry** | Desktop/Laptop computer | E-Mail | PDA | Pager | Project staff | Existing non-prescribing staff | Other Methods | **Trainees** | **Physicians** | **Advanced Practice Nurses** | **Physician Assistants** | **Pharmacists** | **Other health professionals** | Pilot tested | Users trained | Feedback at time of care | CCDSS suggested diagnoses/ treatments/procedures | Authors as developers |
| Peck, 1973 [4] | **+** | **-** | **-** | ? | ? | ? | ? | ? | **-** | **-** | **-** | **+** | **-** | **-** | + | - | - | - | - | - | - | **+** | **+** | **-** | **-** | **-** | **-** | - | - | + | + | - |
| McDonald, 1976 [5] | **-** | **+** | **-** | ? | ? | ? | ? | ? | **+** | **+** | **+** | **-** | **-** | **-** | - | - | - | - | - | + | - | **+** | **+** | **-** | **-** | **-** | **-** | - | - | + | + | + |
| Rodman, 1984 [6] | **+** | **-** | **-** | ? | ? | ? | ? | ? | **-** | **-** | **-** | **+** | **-** | **-** | - | - | - | - | - | + | - | **-** | **+** | **-** | **-** | **+** | **-** | + | + | + | + | + |
| White, 1984 [7] | **-** | **+** | **-** | ? | ? | ? | ? | ? | **+** | **-** | **-** | **-** | **-** | **-** | - | - | - | - | - | + | - | **+** | **+** | **-** | **-** | **-** | **-** | - | + | + | + | + |
| Hurley, 1986 [8] | **+** | **-** | **-** | - | ? | ? | ? | ? | **-** | **-** | **+** | **-** | **-** | **-** | - | - | - | - | + | - | - | **-** | **+** | **-** | **-** | **-** | **-** | + | - | ~ | + | - |
| Carter, 1987 [9] | **+** | **-** | **-** | ? | ? | ? | ? | ? | **-** | **-** | **+** | **-** | **-** | **-** | - | - | - | - | + | - | - | **+** | **+** | **-** | **-** | **-** | **-** | + | + | ~ | + | - |
| White, 1987 [10] | **+** | **-** | **-** | ? | ? | ? | ? | ? | **-** | **-** | **+** | **+** | **-** | **-** | + | - | - | - | - | - | - | **+** | **+** | **-** | **-** | **+** | **-** | - | + | + | + | + |
| Begg, 1989 [11] | **+** | **-** | **-** | ? | ? | ? | ? | ? | **-** | **-** | **-** | **+** | **-** | **-** | ? | ? | ? | ? | ? | ? | ? | **-** | **+** | **-** | **-** | **-** | **-** | - | - | + | + | + |
| Gonzalez, 1989 [12] | **+** | **-** | **-** | ? | ? | ? | ? | ? | **?** | **?** | **?** | **?** | **?** | **?** | ? | ? | ? | ? | ? | ? | ? | **+** | **+** | **-** | **-** | **-** | **-** | ? | + | + | + | - |
| Hickling, 1989 [13] | **+** | **-** | **-** | ? | ? | ? | ? | ? | **?** | **?** | **?** | **?** | **?** | **?** | ? | ? | ? | ? | ? | ? | ? | **-** | **+** | **-** | **-** | **-** | **-** | - | - | + | + | + |
| Burton, 1991 [14] | **+** | **-** | **-** | ? | ? | ? | ? | ? | **?** | **?** | **?** | **?** | **?** | **?** | ? | ? | ? | ? | ? | ? | ? | **+** | **+** | **-** | **-** | **-** | **-** | - | - | + | + | - |
| White, 1991 [15] | **+** | **-** | **-** | ? | ? | ? | ? | ? | **-** | **-** | **-** | **+** | **-** | **-** | + | - | - | - | - | - | - | **-** | **+** | **-** | **-** | **-** | **-** | - | - | - | + | - |
| Ryff-de Lèche, 1992 [16] | **+** | **-** | **-** | ? | ? | ? | ? | ? | **-** | **-** | **-** | **-** | **-** | **+** | + | - | - | - | - | - | - | **-** | **+** | **-** | **-** | **-** | **-** | + | - | + | + | + |
| Casner, 1993 [17] | **+** | **-** | **-** | ? | ? | ? | ? | ? | **-** | **-** | **-** | **+** | **-** | **-** | - | - | - | - | + | - | - | **-** | **+** | **-** | **-** | **-** | **-** | - | + | + | + | - |
| Poller, 1993 [18] | **+** | **-** | **-** | ? | ? | ? | ? | ? | **-** | **-** | **-** | **+** | **-** | **-** | + | - | - | - | - | - | - | **-** | **+** | **-** | **-** | **-** | **-** | - | - | + | + | - |
| Fihn, 1994 [19] | **+** | **-** | **-** | ? | ? | ? | ? | ? | **-** | **+** | **-** | **+** | **-** | **-** | ? | ? | ? | ? | ? | ? | ? | **-** | **+** | **-** | **-** | **-** | **-** | - | + | + | + | + |
| Fitzmaurice, 1996 [20] | **+** | **-** | **-** | ? | ? | ? | ? | ? | **-** | **-** | **+** | **-** | **-** | **-** | - | - | - | - | - | + | - | **-** | **-** | **+** | **-** | **-** | **+** | + | + | - | + | - |
| Overhage, 1997 [21] | **-** | **+** | **+** | ? | ? | ? | ? | ? | **+** | **-** | **-** | **+** | **-** | **-** | + | - | - | - | - | - | - | **+** | **+** | **-** | **-** | **-** | **-** | - | - | + | + | + |
| Vadher, 1997 [22] | **+** | **-** | **-** | ? | ? | ? | ? | ? | **-** | **+** | **-** | **-** | **-** | **-** | - | - | - | - | + | - | - | **-** | **-** | **+** | **-** | **-** | **-** | + | - | + | + | + |
| Ageno, 1998 [23] | **+** | **-** | **-** | ? | ? | ? | ? | ? | **-** | **-** | **+** | **-** | **-** | **-** | + | - | - | - | - | - | - | **-** | **+** | **-** | **-** | **-** | **-** | - | - | + | + | - |
| Poller, 1998 [24] | **+** | **-** | **-** | ? | ? | ? | ? | ? | **-** | **-** | **-** | **+** | **-** | **-** | + | - | - | - | - | - | - | **-** | **+** | **-** | **-** | **-** | **-** | + | + | + | + | - |
| Fitzmaurice, 2000 [25] | **+** | **-** | **-** | ? | ? | ? | ? | ? | **-** | **-** | **-** | **-** | **-** | **+** | + | - | - | - | - | - | - | **-** | **-** | **-** | **-** | **-** | **+** | + | + | + | + | - |
| Manotti, 2001 [26] | **-** | **+** | **-** | ? | ? | ? | ? | ? | **+** | **-** | **-** | **+** | **-** | **-** | + | - | - | - | - | - | - | **-** | **+** | **-** | **-** | **-** | **-** | + | - | + | + | + |
| Claes, 2005 [27, 28] | **+** | **-** | **-** | ? | ? | ? | ? | ? | **-** | **+** | **-** | **-** | **-** | **+** | - | - | - | - | - | - | + | **-** | **+** | **-** | **-** | **-** | **-** | ? | + | - | + | ? |
| Mitra, 2005 [29] | **+** | **-** | **-** | ? | ? | ? | ? | ? | **?** | **?** | **?** | **?** | **?** | **?** | ? | ? | ? | ? | ? | ? | ? | **-** | **+** | **-** | **-** | **-** | **-** | ? | ? | + | + | ? |
| Rood, 2005 [30] | **-** | **+** | **?** | + | + | ? | ? | + | **+** | **-** | **-** | **-** | **-** | **-** | + | - | - | - | - | - | - | **-** | **+** | **-** | **-** | **-** | **+** | + | + | + | + | + |
| Tierney, 2005 [31] | **-** | **+** | **+** | + | + | ? | ? | + | **+** | **-** | **-** | **+** | **-** | **-** | + | - | - | - | - | - | + | **+** | **+** | **-** | **-** | **+** | **-** | ? | + | + | + | + |
| Judge, 2006 [32] | **-** | **+** | **+** | ? | - | - | - | + | **-** | **+** | **-** | **-** | **-** | **-** | + | - | - | - | - | - | - | **-** | **+** | **+** | **+** | **-** | **-** | - | - | + | + | + |
| Albisser, 2007 [33] | **+** | **-** | **-** | + | + | + | ? | ? | **-** | **-** | **-** | **-** | **+** | **-** | + | - | - | - | - | - | - | **-** | **+** | **-** | **-** | **-** | **-** | + | + | - | + | + |
| Matheny, 2008 [34] | **-** | **+** | **-** | + | - | - | - | + | **+** | **-** | **-** | **-** | **-** | **-** | + | - | - | - | - | - | - | **-** | **+** | **-** | **-** | **-** | **-** | + | + | + | + | + |
| Poller, 2008 [35-37] | **+** | **-** | **-** | ? | ? | ? | ? | ? | **?** | **?** | **?** | **?** | **?** | **?** | ? | ? | ? | ? | ? | ? | ? | **-** | **+** | **-** | **-** | **-** | **-** | ? | + | + | + | ? |
| Saager, 2008 [38] | **+** | **-** | **-** | ? | ? | ? | ? | ? | **?** | **?** | **?** | **?** | **?** | **?** | ? | ? | ? | ? | ? | ? | ? | **-** | **-** | **-** | **-** | **-** | **+** | ? | ? | + | + | ? |
| Cavalcanti, 2009 [39] | **+** | **-** | **-** | + | ? | ? | ? | ? | **-** | **-** | **-** | **-** | **-** | **+** | + | - | + | - | - | - | - | **-** | **-** | **-** | **-** | **-** | **+** | + | + | + | + | + |

Abbreviations: CCDSS, computerized clinical decision support system; CPOE, computerized order entry system; EMR, electronic medical record; PDA, personal digital assistant.

^a^Symbol key: + = characteristic present; - = characteristic absent; ~ = characteristic sometimes present; ? = unstated or uncertain.

.
