# Supplementary material for: Computerized clinical decision support systems for therapeutic drug monitoring and dosing: A decision-maker-researcher partnership systematic review
Source: Implement Sci. 2011 Aug 3;6:90. doi: 10.1186/1748-5908-6-90 (PMC3170236; doi:10.1186/1748-5908-6-90)
Supplement: Additional file 3 — Study characteristics for trials of therapeutic drug monitoring and dosing. Study characteristics of the included studies. [file 1748-5908-6-90-S3.DOCX]

**Additional file 3, Table S3. Study characteristics for trials of therapeutic drug monitoring and dosing^a^**

| **Study (Country)** | **Methods Score^b^** | **Funding Source** | **Indication** | **No. of Practitioners / Patients** | **Setting^c^ (No. of clinics / sites)** | **CCDSS Intervention** | **Comparison** |
| --- | --- | --- | --- | --- | --- | --- | --- |
| **Warfarin and Oral Anticoagulant Dosing** | | | | | | | |
| Poller, 2008 [35-37], Various | 5 | Public, Private | Oral anticoagulant therapy initiation and maintenance in outpatients receiving anticoagulation for AF, DVT or PE, mechanical heart valves, or other indications. | 69 / 13219 | •Academic centre •Hospital outpatients (32/32) | 1 of 2 CCDSSs (PARMA or DAWN AC) determined appropriate oral anticoagulant dosing (warfarin, acenocoumarol, or phenprocoumon) to maintain INR within target range and date for next patient visit. Both programs had separate algorithms for induction dosing vs maintenance or steady-state dosing. Computer decisions were reviewed by an experienced physician at each visit. | Usual care |
| Claes, 2005 [27, 28], Belgium | 6 | Private | Oral anticoagulation therapy dosing for outpatients receiving anticoagulation for AF, DVT, PE, mechanical prosthetic heart valve, antiphospholipid syndrome, or to prevent arterial thromboembolism. | 96 / 834 | •Other •Primary care •In a solo practice (66/66) | All physicians received multifaceted education. (Group 1) DAWN AC computer assisted advice provided dosing and visit recommendations based on patients’ INR values. Advice was faxed by pathologist to physician the afternoon blood was drawn. Physicians could follow or ignore advice. | (Group 2) CoaguChek device provided on the spot INR values in clinic. (Group 3) Physicians received feedback on INR management performance every 2 months. (Group 4) Control group physicians received multifaceted education. |
| Mitra, 2005 [29], USA | 5 | ... | Warfarin dosing in hospitalised rehabilitation patients. | ... / 30 | •Other •Academic centre •Hospital inpatients (1/1) | CCDSS (DAWN AC) provided instructions to physicians for warfarin dosing and timing and frequency of blood draws to maintain a target INR of 2.0 to 3.0. | Usual care |
| Manotti, 2001 [26], Italy | 4 | ... | Oral anticoagulation therapy maintenance for outpatients receiving anticoagulation for VTE, non-ischemic heart disease, arterial disease, heart-valve prosthesis, and other diagnoses. | ... / 1251 | •Academic centre (5/...) | CCDSS (PARMA) used an algorithm based on patient demographic, clinical, and follow-up data, to suggest oral anticoagulant doses and follow-up appointments. | Usual care |
| Fitzmaurice, 2000 [25], UK | 6 | Public | Warfarin maintenance for outpatients with a range of indications including AF, DVT, PE or SE, arterial disease, mechanical prosthetic valves, transient ischemic attack or cerebrovascular accident, cardiomyopathy, mitral or aortic stenosis, coronary artery bypass, or heart valve replacement. | ... / 367 | •Primary care (12/...) | CCDSS recommended warfarin dosing based on patient INR and individual therapeutic range in nurse-led clinic. Recommendations could be overridden. | Usual care |
| Ageno, 1998 [23], Canada | 6 | ... | Warfarin maintenance for outpatients with mechanical heart valves. | ... / 101 | •Academic centre •Hospital outpatients (1/1) | CCDSS (DAWN AC) recommended INR dosing and next visit date for individual patients based on latest INR, target INR (3.0 according to local practice), and prespecified dose change rules. Clinicians could override the dose and visit intervals if needed. System did not dose for INR >5.0. | Standard manual INR monitoring and dosing by 2 physicians and 3 registered nurses |
| Poller, 1998 [24], UK, Denmark, Portugal, Norway | 3 | Public | Anticoagulant therapy initiation and maintenance for outpatients. | ... / 285 | •Academic centre •Hospital outpatients (5/5) | CCDSS (DAWN AC) generated anticoagulant dosing schedules and time to next INR test using 2 main modules. The induction module was for dosing initial warfarin therapy over the first 4 days to reach a dose within 1 mg of eventual maintenance dose. The maintenance module adjusted the dose to reach and sustain the therapeutic range. | Usual care |
| Vadher, 1997 [22], UK | 6 | Public | Warfarin initiation and maintenance for inpatients and outpatients with DVT, PE or SE, AF, valve disease, or mural thrombus, or who needed prophylaxis. | 49 / 148 | •Academic centre •Hospital inpatients (1/1) | CCDSS used simple proportional-derivative control methods to provide recommendations for initial and maintenance dosing of oral anticoagulation. Maintenance dosing was based on previous dose and difference between target and actual INR. Physicians could choose to accept or reject dosing recommendations, and also received guidelines on anticoagulation. | Usual care plus guidelines on anticoagulation |
| Fitzmaurice, 1996 [20], UK | 4 | ... | Warfarin maintenance for outpatients for DVT, PE or SE, AF, mechanical prosthetic heart valve, recurrent DVT and PE, and prevention of thromboembolism in myocardial infarction. | ... / 49 | •Academic centre •Hospital outpatients •Primary care (2/1) | CCDSS recommended warfarin dosing or warfarin suspension and time to next visit based on patient INR. | Usual care |
| Fihn, 1994 [19], USA | 3 | Public, Private | Frequency of warfarin monitoring in outpatients. | ... / 849 | •Academic centre •Primary care •Community-based clinic (5/5) | CCDSS generated recommendations for scheduling patient follow-up visits for physicians at the anticoagulation clinic. Recommendations were based on patient data and physician-selected prothombin time ratio (PTR)/INR targets. Physicians were allowed to disregard or modify the scheduling recommendations as well as reweight or discount a patient’s past history of prothrombin time ratio. | Usual care |
| Poller, 1993 [18], UK | 5 | ... | Warfarin maintenance and dosing for outpatients who started anticoagulation for VTE; arterial, heart, or cerebrovascular disease; lone AF; rheumatic heart disease; or another disorder. | ... / 186 | •Academic centre •Subspecialty clinic  (1/1) | 2 CCDSSs: (A) Charles Anticoagulant Clinic Manager, and (B) Coventry program suggested warfarin doses or warfarin suspension and interval to next clinic visit based on patient INR values. Note: Hillingdon system was discontinued during study and is not included in this review. | (C) Usual dosing by experienced medical staff in anticoagulant clinic |
| White, 1991[15], USA | 6 | ... | Warfarin maintenance and dosing for outpatients on long-term warfarin therapy. | ... / 50 | •Academic centre •Hospital outpatients (1/1) | CCDSS used Bayesian forecasting methods, pharmacokinetic and pharmacodynamics modelling, and patient data to predict steady-state warfarin dosing needed to reach a target prothrombin time. Nurse-specialists entered warfarin doses and steady-state prothrombin times into the CCDSS. | Usual care with dose adjustments made by nurse-specialist experienced in management of oral anticoagulation |
| Carter, 1987 [9], USA | 2 | Public | Warfarin initiation dosing for hospital inpatients. | ... / 54 | •Academic centre •Hospital inpatients (1/1) | CCDSS suggested warfarin dosages (analog-computer method) or a single dosage prediction was made using a formula (linear-regression method) for adult inpatients. | Usual care |
| White, 1987 [10], USA | 6 | ... | Warfarin initiation and dosing for patients hospitalised with DVT, cerebrovascular accident, transient ischemic attack, PE, or AF. | ... / 75 | •Academic centre •Hospital inpatients (2/...) | CCDSS (Warfcalc) used Bayesian forecasting methods to determine appropriate warfarin dosing based on patient data, including response to warfarin therapy. Warfarin therapy was managed by a physician or pharmacist familiar with the CCDSS but who were not experts in management of warfarin therapy. Primary physicians selected target prothrombin ratio. | Usual care. Physicians selected target prothrombin ratio. |
| **Aminophylline and Theophylline Dosing** | | | | | | | |
| Tierney, 2005 [31], USA | 9 | Public | Management of asthma and COPD in adults in primary care. | 266 / 706 | •Academic centre •Primary care (4/...) | Existing computer workstations were programmed to provide care suggestions to physicians and pharmacists based on evidence-based guidelines for asthma and COPD management and data in patient EMRs. Physicians received CCDSS-generated care suggestions on paper medication lists at patient visits and on computer workstations when writing orders. Pharmacists received them electronically and could choose to do nothing, or discuss suggestions with patients or physicians. They received same educational material as the control group. | Physicians and pharmacists received a printed summary of asthma and COPD management guidelines and could attend rounds about the guidelines but did not receive care suggestions. |
| Casner, 1993 [17], USA | 3 | ... | Theophylline dosing for inpatients with asthma or COPD. | ... / 47 | •Hospital inpatients (1/1) | Pharmacokinetic CCDSS (linear one-compartment model) was used to predict theophylline infusion rates to achieve a target serum level of 15 mg/L. The CCDSS was run on hand-held computers and adjusted dosing based on 2 early measures of serum theophylline levels. | Physician-directed theophylline infusion adjustments to achieve a target serum level of 15 mg/L based on 2 early serum measures |
| Gonzalez, 1989 [12], USA | 6 | Private | Drug-dosing of aminophylline in the emergency department. | ... / 67 | •Academic centre •Hospital inpatients (.../...) | CCDSS used Bayesian pharmacokinetic model to estimate aminophylline loading and maintenance dosing for individual patients to achieve serum theophylline levels of 15 mg/L (12 mg/L if oral theophylline given within 6h). | Dosing nomogram based on emergency department asthma guidelines and theophylline level 4 hours after initial bolus were used to determine aminophylline dosing needed to maintain a serum theophylline level of 10-20 mg/L. |
| Hurley, 1986 [8], Australia | 8 | Public, Private | Theophylline dosing for inpatients with acute air-flow obstruction. | ... / 96 | •Hospital inpatients (1/1) | Initial loading and infusion doses of theophylline were based on a nomogram; subsequent infusion and oral doses were adjusted based on CCDSS pharmacokinetic analysis of theophylline serum levels. | Physicians selected IV and oral doses of theophylline based on drug serum levels without use of formal pharmacokinetic analysis. |
| **Insulin Dosing and Glucose / Glycaemic Regulation** | | | | | | | |
| Cavalcanti, 2009 [39], Brazil | 8 | Public, Private | Glucose measurement and insulin dosing for glucose control for ICU patients. | 60 / 168 | •Academic centre •Hospital inpatients (5/5) | CCDSS (computer assisted insulin protocol, [CAIP]) used patient data including current infusion rate, glucose level and time between previous glucose measurements to make recommendations for intravenous insulin dosing and glucose monitoring to maintain a blood glucose between 100 and 130 mg/dL. The CCDSS was available via desktop or handheld computers for nursing staff at hospital based ICUs. The nurses input patient data and followed the recommendations provided. Recommendations were determined by the authors who created the algorithms. | Leuven: A strict glycaemic control protocol for intravenous insulin infusion with target blood glucose between 80 and 110 mg/dL. All insulin adjustments were made by nurses. Conventional: Subcutaneous insulin is administered for blood glucose levels > 150 mg/dL according to a sliding scale. All insulin adjustments were made by nurses. |
| Saager, 2008 [38], USA | 6 | Public | Glucose management in diabetic patients in cardiothoracic ICUs. | ... / 40 | •Academic centre •Hospital inpatients (1/1) | CCDSS (EndoTool Glucose Management System) recommended insulin dose, glucose determination frequency, and a 50% dextrose dose (when appropriate) for hypoglycaemia, based on blood glucose readings from a point-of-care device. It uses the previous 4 dose responses to regulate the dosing relationship, and is designed to be used by trained health care professionals. | Standard locally-developed paper-based ICU insulin protocol with target blood sugar levels between 90 and 150 mg/dL |
| Albisser, 2007 [33], USA | 8 | ... | Prediction of glycaemia and risk for hypoglycaemia in insulin-dependent patients in primary care. | 2 / 22 | •Subspecialty clinic  •Primary care (.../...) | CCDSS predicted individual patient glycaemia and risks for hypoglycaemia based on daily patient reports of self-measured blood glucose and life-style factors. Patients entered data into a database shared with providers through the Internet or by telephone, through an interactive voice response system. During remote, weekly, telemedical interventions, providers accessed the shared database using a graphical user interface to review the risks displayed on-screen. | Providers in the control group used the same interface as the intervention group for remote, weekly, telemedical interventions but did not have access to the risk predictions. |
| Rood, 2005 [30], The Netherlands | 8 | ... | Management of glucose regulation in critically ill patients. | 104 / 484 | •Other •Hospital inpatients (1/1) | CCDSS monitored the interval between glucose measurements and made guideline-based recommendations for timing between glucose measurements and administration of insulin doses in ICU patients. Recommendations were displayed electronically in pop-up windows when patient records were activated. | Paper-based guideline for timing between glucose measurements and administration of insulin doses in ICU patients |
| Ryff-de Lèche, 1992 [16], Switzerland | 3 | Private | Insulin dosing for diabetes management in outpatients. | ... / 38  19 in each study | •Hospital outpatients (1/1) | In this 3-month crossover study, a commercially available CCDSS (Camit S1) analyzed and summarized blood glucose data that was measured and entered by patients in an electronic log book. Diabetologists reviewed the CCDSS results at 3-wk intervals (without seeing the patients) and sent therapy recommendations to patients. Note: A 2^nd^ study reported in this article compared 2 CCDSSs and was not eligible for the review. | Diabetologists recommended insulin doses at 3-week intervals based on patient’s paper log-books. |
| McDonald, 1976 [5], USA | 2 | ... | Use of laboratory tests to detect potential medication-related events in adults attending a diabetes clinic. | ... / 226 | •Academic centre •Subspecialty clinic  (1/1) | CCDSS generated protocol-driven recommendations for repeat laboratory tests and treatment changes based on EMR data, including past lab results, medications prescribed, and time since previous tests. Recommendations were printed as part of patient reports and placed at the front of patient charts before visits. | Usual care |
| **Aminoglycoside Dosing** | | | | | | | |
| Burton, 1991 [14], USA | 6 | Public | Aminoglycoside dosing for inpatients with clinical infections. | ... / 147 | •Academic centre •Hospital inpatients (1/1) | CCDSS with Bayesian-based algorithm used serum aminoglycoside level data to predict aminoglycoside dosage needed to achieve peak (gentamicin and tobramycin, 5-10 mg/L; amikacin, 20-30 mg/L) and trough (gentamicin and tobramicin, <2mg/L; amikacin, <5mg/L) target levels. | Physician-directed aminoglycoside dosing using serum level data |
| Begg, 1989 [11, New Zealand | 5 | ... | Individualised aminoglycoside dosing for inpatients receiving gentamicin or tobramycin. | ... / 50 | •Hospital inpatients (.../...) | CCDSS used pharmacokinetic analysis (one-compartment model) to predict individualised aminoglycoside doses and dose intervals needed to achieve a peak level at end of infusion of 8 mg/L and trough level of 1.5 mg/L. | Routine clinical practice used to achieve peak plasma aminoglycoside levels of 6-10 mg/L and trough levels of 1-2 mg/L. |
| Hickling, 1989 [13], New Zealand | 3 | Private | Pharmacokinetic dosage prediction for aminoglycosides based on estimated creatinine clearance in critically ill patients. | ... / 32 | •Hospital inpatients (1/1) | CCDSS pharmacokinetic model was used to predict early therapeutic dose and dose interval of aminoglycoside to achieve any desired peak and trough concentration in critically ill patients, based on 3 post-distributional plasma concentrations after the initial dose. | Nomogram was used to determine the aminoglycoside dose and dose interval based on estimated creatinine clearance |
| **Digoxin Dosing / Monitoring** | | | | | | | |
| White, 1984 [7], USA | 4 | ... | Monitoring signs and risk factors for digoxin intoxication in inpatients. | ... / 396 | •Academic centre •Hospital inpatients (1/1) | CCDSS (Health Evaluation through Logical Processing [HELP]) accessed a clinical patient database nightly and used expert-determined decision criteria to identify concerns (drug interactions or signs of potential digoxin intoxication) for patients taking digoxin. Concerns were summarized in alert reports placed in patient charts. | Usual care |
| Peck, 1973 [4], USA | 6 | Public | Digoxin dosing recommendations for outpatients with congestive heart failure. | 4 / 42 | •Academic centre •Hospital outpatients •Subspecialty clinic  (1/1) | CCDSS used patient data, including a measure of renal function, and physician objectives to provide a digoxin dosing scheme that would achieve a desired steady-state serum digoxin level. Physicians could choose to accept or reject the computer-provided dosing scheme. | Usual care |
| **Lidocaine Dosing** | | | | | | | |
| Rodman, 1984 [6], USA | 6 | Public | Lidocaine dosing for patients in ICUs or coronary care units. | ... / 20 | •Academic centre •Hospital inpatients (1/1) | CCDSS recommended lidocaine infusion regimen based on patient’s age, sex, height, weight, cardiac index, past lidocaine therapy, and desired lidocaine concentration for ICU and coronary care unit patients. | Usual care |
| **Miscellaneous** | | | | | | | |
| Matheny, 2008 [34], USA | 8 | Public | Routine medication laboratory monitoring in primary care. | 303 / 1922 | •Academic centre •Hospital outpatients •Primary care •Community-based clinic patients (20/20) | CCDSS-generated reminders for laboratory testing (potassium, creatinine, liver or thyroid function, and therapeutic drug levels) appeared on EHRs during visits of patients who were on an included medication for ≥ 365 days with no relevant laboratory test in the past 365 days. | Usual care |
| Judge, 2006 [32], USA | 8 | Public | Safety of medication prescribing in a long-term care setting. | 27 / 445 | •Academic centre •Long term care (includes nursing home) (1/1) | CCDSS displayed evidence-based real-time alerts in a pop-up box on the CPOE system when prescribers entered drug orders that posed a potential risk, required monitoring for adverse events, or needed action to prevent adverse events. The 41 potential alerts were informational and did not require specific actions. | Alerts generated but not displayed to prescribers |
| Overhage, 1997 [21], USA | 8 | Public | Identification of corollary orders to prevent errors of omission for tests and treatments in hospital inpatients on a general medicine ward. | 92 / 2181 | •Academic centre •Hospital inpatients (1/1) | A rule-based reminder CCDSS determined corollary orders for 87 target orders and displayed these on-line to physicians using the CPOE. Corollary orders could be accepted or rejected by physicians. | Physicians used CPOE but did not receive on-line corollary orders |

Abbreviations: AF, atrial fibrillation; CCDSS, computerized clinical decision support system; COPD, chronic obstructive pulmonary disease; CPOE, computerized order entry system; DVT, deep vein thrombosis; EMR, electronic medical record; EHR, electronic health record; GUSTO, Global Utilization of Streptokinase and TPA for Occluded coronary arteries); ICU, intensive care unit; INR, international normalised ratio; IV, intravenous; PARMA, Program for Archive, Refertation and Monitoring of Anticoagulated patients; PE, pulmonary embolism; SE, systemic embolism; TPA, tissue plasminogen activator; VTE, venous thromboembolism.

^a^Ellipses (…) indicate item was not assessed.

^b^Based on 5 individual items (score 2 = yes, 1 = partly, and 0 = No) and a summed total score (range 0 to 10). Because this review update included only randomized, controlled trials, the total score differs from that reported in the previous version of this review [1]: the item evaluating study type (randomized, quasi-randomized, or concurrent controls) has been replaced by one that evaluates use of concealed allocation (concealed, unclear, not concealed).

^c^Diabetes clinic is an example of a subspecialty clinic.

.
