# Supplementary material for: Computerized clinical decision support systems for therapeutic drug monitoring and dosing: A decision-maker-researcher partnership systematic review
Source: Implement Sci. 2011 Aug 3;6:90. doi: 10.1186/1748-5908-6-90 (PMC3170236; doi:10.1186/1748-5908-6-90)
Supplement: Additional file 4 — Results for CCDSS trials of therapeutic drug monitoring and dosing. Details results of the included studies. [file 1748-5908-6-90-S4.DOCX]

**Additional file 4, Table S4. Results for CCDSS trials of therapeutic drug monitoring and dosing^a^**

| **Study** | **Process of care outcomes** | **CCDSS vs control data** | **Patient outcomes** | **CCDSS vs control data** | **CCDSS process of care effect**^b^ | **CCDSS patient effect**^b^ |
| --- | --- | --- | --- | --- | --- | --- |
| **Warfarin and Oral Anticoagulant Dosing** | | | | | | |
| Poller, 2008 [35-37] | **Secondary 1. Mean (SD) % time INR in range during 4.5 year study; difference (95% CI), adjusted for computer program, gender, age, clinical indication, and target INR difference.**  **1a. All weeks.**  1b. Weeks 1-3.  1c. Weeks 4-9.  1d. Weeks 10-21.  1e. Weeks 22+.   Planned subgroup analysis by clinical indication. 2. Mean (SD) % time INR in range during 4.5 year study:  2a. Atrial fibrillation.  2b. Deep vein thrombosis/pulmonary embolism.  2c. Mechanical heart valves.  2d. Other indication.   Supplementary article reported data for subgroup PARMA vs control during 4.5 year study. 3. Mean (SD) % time INR in range; difference (95% CI) adjusted for computer program, gender, age, clinical indication, and target INR difference.  3a. All weeks.  3b. Weeks 1-3.  3c. Weeks 4-9.  3d. Weeks 10-21.  3e. Weeks 22+.  4. Mean % time INR below range.  4a. All weeks.  4b. Weeks 1-3.  4c. Weeks 4-9.  4d. Weeks 10-21.  4e. Weeks 22+.  5. Mean % time INR above range.  5a. All weeks.  5b. Weeks 1-3.  5c. Weeks 4.9.  5d. Weeks 10-21.  5e. Weeks 22+.  6. Mean % time INR at 2-4.5.  6a. All weeks.  6b. Weeks 1-3.  6c. Weeks 4-9.  6d. Weeks 10-21.  6e. Weeks 22+.  7. Mean (SD) INR.  7a. All weeks.  7b. Weeks 1-3.  7c. Weeks 4-9.  7d. Weeks 10-21.  7e. Weeks 22+.  Supplementary article reported data for subgroup DAWN-AC vs control during 4.5 year study. 8. Mean (SD) % time INR in range; difference (95% CI) adjusted for computer program, gender, age, clinical indication, and target INR difference.  8a. All weeks.  8b. Weeks 1-3.  8c. Weeks 4-9.  8d. Weeks 10-21.  8e. Weeks 22+.  9. Mean % time INR below range.  9a. All weeks.  9b. Weeks 1-3.  9c. Weeks 4-9.  9d. Weeks 10-21.  9e. Weeks 22+.  10. Mean % time INR above range.  10a. All weeks.  10b. Weeks 1-3.  10c. Weeks 4-9.  10d. Weeks 10-21.  10e. Weeks 22+.  11. Mean % time INR at 2-4.5.  11a. All weeks.  11b. Weeks 1-3.  11c. Weeks 4-9.  11d. Weeks 10-21.  11e. Weeks 22+.  12. Mean (SD) INR.  12a. Over all weeks.  12b. Weeks 1-3.  12c. Weeks 4-9.  12d. Weeks 10-21.  12e. Weeks 22+.  Note: Figure 3 in main and supplementary papers show results by clinical centre. | 1a. 65.9% (16.5) vs 64.7% (17.0), 1.2% (0.7 to 1.8), *P<*.001  1b. 49.3% vs 49.3%  1c. 56.5% vs 55.9%  1d. 63.2% vs 62.0%  1e. 68.9% vs 67.4%  2a. 67.6 (15.7) vs 66.2 (16.1), *P*=NR  2b. 66.0 (17.7) vs 64.9 (17.6), *P*=NR  2c. 62.5 (16.0) vs 62.6 (16.9), *P*=NR  2d. 63.7 (17.1) vs 61.5 (18.7), *P*=NR  3a. 65,7% (16.5) vs 65.0% (16.9); 0.7% (0.1 to 1.3), *P=*.021  3b. 48.6% (32.6) vs 48.9% (32.0)  3c. 55.5% (33.8) vs 55.5% (32.7)  3d. 62.5% (28.3) vs 61.6% (28.5)  3e. 68.8% (15.7) vs 67.7% (16.7)  4a. 22.3% vs 22.9%  4b. 35.9% vs 36.7%  4c. 34.0% vs 33.1%  4d. 26.8% vs 26.9%  4e. 19.1% vs 20.1%  5a. 11.9% vs 12.1%  5b. 15.5% vs 14.5%  5c. 10.5% vs 11.5%  5d. 10.7% vs 11.5%  5e. 12.0% vs 12.1%  6a. 80.0% vs 79.9%  6b. 65.5% vs 64.7%  6c. 68.8% vs 69.7%  6d. 75.8% vs 76.1%  6e. 83.1% vs 82.8%  7a. 2.48 (0.88) vs 2.47 (0.85)  7b. 2.36 (1.17) vs 2.35 (1.10)  7c. 2.36 (0.87) vs 2.36 (0.85)  7d. 2.43 (0.81) vs 2.44 (0.84)  7e. 2.52 (0.82) vs 2.52 (0.79)  8a. 66.8% (16.4) vs 63.4% (17.7); 3.5% (2.3 to 4.9), *P<*.001  8b. 51.7% (34.6) vs 51.1% (33.6)  8c. 60.7% (31.8) vs 58.4% (33.7)  8d. 66.2% (27.6) vs 62.9% (29.5)  8e. 69.6% (16.2) vs 64.4% (17.2)  9a. 19.7% vs 21.1%  9b. 32.7% vs 32.5%  9c. 25.3% vs 27.3%  9d. 20.4% vs 22.3%  9e. 17.7% vs 21.4%  10a. 13.5% vs 15.5%  10b. 15.5% vs 16.4%  10c. 14.0% vs 14.3%  10d. 13.4% vs 14.7%  10e. 12.7% vs 14.2%  11a. 82.4% vs 79.2%  11b. 68.1% vs 68.5%  11c. 76.7% vs 74.1%  11d. 81.7% vs 78.6%  11e. 84.7% vs 81.2%  12a. 2.49 (0.94) vs 2.48 (1.00)  12b. 2.29 (1.15) vs 2.30 (1.22)  12c. 2.45 (1.27) vs 2.44 (0.88)  12d. 2.49 (0.89) vs 2.51 (0.89)  12e. 2.54 (0.81) vs 2.53 (0.99) | Patients/patient-years analyzed: 6605/9353 vs 6447/9264  **Primary outcome:  1. Number of adjudicated clinical events / events per 100 patient-years; adjusted incidence rate ratio (95% CI).**  **1a. Overall.**  1b. In patients 1^st^ 3 weeks of study.  2. Number of minor bleeds / events per 100 patient-years.  3. Number of major bleeds / events per 100 patient-years.  4. Number of thrombotic events / events per 100 patient-years.  5. Number of deaths; number per 100 patient-years.  6. Number of fatal bleeds during 4.5 year study.  7. Number of fatal thrombotic events during 4.5 year study.  Planned subgroup analysis by clinical indication: 8. Number of adjudicated clinical events / events per 100 patient-years; incidence rate ratio (95% CI) adjusted for gender, age at entry, clinical indication, and target INR range (<1 favours treatment): 8a. Atrial fibrillation.  8b. Deep vein thrombosis/pulmonary embolism.  8c. Mechanical heart valves.  8d. Other indication.  8e. Overall interaction.  Subgroup analysis (not clear preplanned)  9. Number of clinical events / events per 100 patient years by INR target range:  9a. Target 2-3 or lower range.  9b. Target 2.5-3.5 or higher range  Subgroup analysis (not clear preplanned).  10. Number of events / events per 100 patient years by patient type.  10a. New patients.  10b. Patients established on oral anticoagulants.  Supplementary article reported data for subgroup PARMA vs control: 11. Number of adjudicated clinical events (bleeding or thrombosis) / events per 100 patient-years; adjusted incidence rate ratio (95% CI).  12. Number of minor bleeds / events per 100 patient-years.  13. Number of major bleeds / events per 100 patient-years.  14. Number of thrombotic events / events per 100 patient-years.  15. Number of deaths; number per 100 patient-years.   Planned subgroup analysis by clinical indication: 16. Number of clinical events / events per 100 patient-years; incidence rate ratio (95% CI) (<1 favours treatment): 16a. Atrial fibrillation.  16b. Deep vein thrombosis/pulmonary embolism.  16c. Mechanical heart valves.  16d. Other indication.  16e. Overall interaction.  Subgroup analysis (not clear preplanned)  17. Number of clinical events / events per 100 patient years by INR target range:  17a. Target 2-3 or lower range.  17b. Target 2.5-3.5 or higher range.  Subgroup analysis (not clear preplanned).  18. Number of events / events per 100 patient years by patient type.  18a. New patients.  18b. Patients established on oral anticoagulants.  19. Number of events in 2542 patients (1322 vs 1220) with deep vein thrombosis/pulmonary embolism.  19a. All deaths.  19b. Fatal bleeds.  19c. Fatal thrombosis.  19d. Other deaths.  19e. Major bleeds.  19f. Minor bleeds.  19g. Thrombotic events.  19h. During 1^st^ 3 weeks.  19i. After week 3.  19j. Total.  Supplementary article reported data for subgroup DAWN-AC vs control:  20. Number of adjudicated clinical events (bleeding or thrombosis) / events per 100 patient-years (95% CI).  21. Number of minor bleeds / events per 100 patient-years.  22. Number of major bleeds / events per 100 patient-years.  23. Number of thrombotic events / events per 100 patient-years.  24. Number of deaths; number per 100 patient-years. 1  Planned subgroup analysis by clinical indication: 25. Number of clinical events / events per 100 patient-years: 25a. Atrial fibrillation.  25b. Deep vein thrombosis/pulmonary embolism.  25c. Mechanical heart valves.  25d. Other indication.  Subgroup analysis (not clear preplanned)  26. Number of clinical events / events per 100 patient years by INR target range:  26a. Target 2-3 or lower range.  26b. Target 2.5-3.5 or higher range  Subgroup analysis (not clear preplanned).  27. Number of events / events per 100 patient years by patient type.  27a. New patients.  27b. Patients established on oral anticoagulants. | 1a. 513 vs 555; 5.5 vs 6.0; 0.90 (0.80 to 1.02), *P=*.10  1b. NR; 8.6 vs 12.3; 0.7 (0.48 to 1.04), *P=*.06  2. 253 vs 288; 2.7 vs 3.1, *P*=NR  3. 93 vs 99; 1.0 vs 1.1, *P*=NR  4. 97 vs 106; 1.0 vs 1.1, *P*=NR  5. 70/6716 vs 62/6503; 0.7 vs 0.7, *P*=NR  6. 9/6716 vs 12/6503, *P*=NR  7. 8/6716 vs 14/6503, *P*=NR  8a. 228 vs 251; 4.9 vs 5.3; 0.93 (0.78 to 1.12)  8b. 115 vs 152; 6.1 vs 9.1; 0.67 (0.52 to 0.85), *P=*.001  8c. 87 vs 83; 6.5 vs 6.1; 1.04 (0.77 to 1.40)  8d. 83 vs 69; 5.5 vs 4.6; 1.20 ( 0.87 to 1.65)  8e. *P=*.02  9a. 402 vs 455; 5.1 vs 5.8  9b. 111 vs 100; 7.6 vs 7.0  10a. 361 vs 397; 5.6 vs 6.4  10b. 152 vs 158; 5.1 vs 5.6  11.420 vs 463; 5.5 vs 6.0; 0.89 (0.78 to 1.01)  12. 211 vs 245; 2.7 vs 3.2, *P*=NR  13. 73 vs 85; 0.9 vs 1.1, *P*=NR  14. 84 vs 85; 1.1 vs 1.1, *P*=NR  15. 52/5377 vs 48/5175; 0.7 vs 0.6, *P*=NR  16a. 172 vs 199; 4.6 vs 5.1, NS  16b. 106 vs 134; 6.7 vs 9.7; 0.69 (0.53 to 0.89), *P=*.005  16c. 78 vs 75; 6.5 vs 6.2, NS  16d. 64 vs 55; 5.4 vs 4.6, NS  16e. *P=*.05  17a. 321 vs 376; 4.9 vs 5.9  17b. 99 vs 87; 8.2 vs 7.0  18a. 292 vs 321; 5.6 vs 6.1  18b. 128 vs 142; 5.1 vs 5.8  19a. 9 vs 10  19b. 1 vs 1  19c. 1 vs 0  19d. 7 vs 9  19e. 11 vs 27  19f. 55 vs 66  19g. 31 vs 31  19h. 15 vs 26  19i. 91 vs 108  19j. 115 vs 152  20. 93 vs 92; 5.6 (4.6 to 6.9) vs 5.8 (4.6 to 7.0)  21. 42 vs 43; 2.5 vs 2.7  22. 23 vs 14; 1.4 vs 0.9  23. 15 vs 23; 0.9 vs 1.4  24. 13/1399 vs 12/1328; 0.8 vs 0.8  25a. 53 vs 51; 6.1 vs 5.9  25b. 9 vs 18; 3.1 vs 6.4  25c. 11 vs 9; 7.3 vs 5.9  25d. 20 vs 14; 6.0 vs 4.6  26a. 81 vs 79; 5.8 vs 5.7  26b. 12 vs 13; 4.8 vs 6.4  27a. 69 vs 76; 5.7 vs 6.2  27b. 24 vs 16; 5.4 vs 4.3 | + | 0 |
| Claes, 2005 [27, 28] | **1. Mean [SE] proportion of time that INR values were within 0.5 INR-units of target range (2.5 or 3.5 depending on indication) during median 4.8 month follow-up (primary outcome). 2. Mean proportion of time that INR values were within 0.75 INR-units of target range (2.5 or 3.5 depending on indication) during median 4.8 month follow-up (primary outcome).** 3. Proportion [SE] of patients with at least 1 INR < 2 (not prespecified). 4. Proportion [SE] of patients with at least 1 INR > 5 (not prespecified)  5. Median number [SE] of tests per patient per month (not prespecified).  6. Proportion of patients [SE] with treatment changes (not prespecified).  7. % change (95% CI) per GP-practice from baseline for target within 0.5 INR units.  8. % change (95% CI) per GP-practice from baseline for target within 0.75 INR units.  Not prespecified  9. Incremental cost-effectiveness (vs usual care); additional cost per day within a 0.5 range from INR target,. | Dawn AC (CCDSS) / CoaguChek / Feedback / Control / Baseline values (p – differences among 4 intervention groups on final values; p’ – overall differences between baseline values and intervention group values, p” – interaction between groups on difference from baseline)  1. 55% [2.3] / 57% [2.2] / 60% [2.2] / 63% [2.5] / 49% [1.4], *P=*.13; *P*’<.001, *P*”=.80  2. 73% [2.3] / 74% [2.2] / 78% [2.3] / 80% [2.4] / 79% [1.4], *P=*.12; *P*’<.001, *P*”=.90  3. 41% [4.3] / 45% [4.1] / 45% [4.3] / 45% [4.6] / 44% [2.2], *P=*.86; *P*’=.67, *P*”=.74  4. 19% [3.4] / 9% [2.2] / 7% [1.8] / 15% [3.1] /21% [1.9], *P=*.009; *P*’=.02, *P*”=.28  5. 1.6 [0.1] / 1.7 [0.1] / 1.7 [0.1] / 1.7 [0.1] / 2 [0.06], *P=*.88; *P<*.001, *P*”=.58  6. 65% [7.7] / 85% [4.4] / 74% [6.4] / 70% [6.9] / NR, *P=*.11  7. 11% (5.5 to 16.5) vs 11% (6 to 16.5) vs 9% (4 to 13.5) vs 8% (2 to 13.5), *P=*.8  8. 12% (6.5 to 17.5) vs 12% (7 to 17) vs 10% (6 to 15) vs 10% (4.5 to 15.5), *P=*.9  9. 4.90 Euros / Dominant (less costly and more effective than usual care) / 5.02 Euros / 5.23 Euros.  Other results are available in supplemental paper. | **1. Number of thromboembolic complications (prespecified secondary outcome) during median 4.8 months follow-up. 2. Number of haemorrhages (prespecified secondary outcome) during median 4.8 months follow-up.** 3. Death from other causes (not prespecified) during median 4.8 months follow-up. | DAWN-AC / CoaguChek / Feedback / Control  1. 3 / 4 / 6 / 4, *P=*.83  2. Minor bleedings 4 / 11/ 14 / 6, *P=*.28  Major bleedings 2 / 5 / 4 / 3, *P=*.78  3. 0 / 3 / 2 / 0, *P=*.09 | 0 | 0 |
| Mitra, 2005 [29] | **1. Proportion of days in therapeutic anticoagulation range (INR 2.0 to 3.0) during hospitalization (primary outcome).**  2. Proportion of time at INR <2.0 during hospitalization (days) (not prespecified).  3. Proportion of time at INR >3.0 during hospitalization (days) (not prespecified). **4. Number (SD) of blood draws (not stated if mean or median) during hospitalization (primary outcome).** | 1. 61.7% vs 44.1%, *P<*.05  2. 20% vs 40%  3. 18% vs 16%  4. 23.3 (7.5) vs 19.5 (10.9), *P=*.170 | **Not prespecified**  **1. Number of patients with incident deep vein thrombosis or pulmonary embolism during hospitalization.**  **2. Mean (SD) length of hospital stay (days).** | 1. 0/14 vs 0/16  2. 38.7 (15.6) vs 31.7 (16.5) | + | … |
| Manotti, 2001 [26] | Long term therapy group (on therapy for followed for ≥ 3 months at enrollment and followed for 1 year) **1. Percentage of time spent by single patients in the scheduled therapeutic range over 1 year (primary outcome)**  **1a. All INR targets.**  **1ai. Overall (744.7 patient/years follow-up).**  1aii. Warfarin patients (519.0 patient/years follow-up).  1aiii. Acenocoumarol patients (255.7 patient/years follow-up).  1b. High target INR.  1bi. Overall.  1bii. Warfarin patients.  1biii. Acenocoumarol patients.  1c. Low target INR.  1ci. Overall.  1cii. Warfarin patients.  1ciii. Acenocoumarol patients.  2. Percentage of time spent by single patients below scheduled therapeutic range over 1 year: high target INR, ≥2.8; low target INR, <2.8.  2a. All INR targets.  2ai. Overall.  2aii. Warfarin patients.  2aiii. Acenocoumarol patients.  2b. High target INR  2bi. Overall.  2bii. Warfarin patients.  2biii. Acenocoumarol patients.  2c. Low target INR  2ci. Overall.  2cii. Warfarin patients.  2ciii. Acenocoumarol patients.  3. Percentage of time spent by single patients above scheduled therapeutic range over 1 year: high target INR, ≥2.8; low target INR, <2.8.  3a. All INR targets.  3ai. Overall.  3aii. Warfarin patients.  3aiii. Acenocoumarol patients.  3b. High target INR  3bi. Overall.  3bii. Warfarin patients.  3biii. Acenocoumarol patients.  3c. Low target INR  3ci. Overall.  3cii. Warfarin patients.  3ciii. Acenocoumarol patients.  Note: In the article, percentage time within, above, and below range is also reported by quarters of the year (separated by drug in Table 4 and by INR target in Table 5).  4. Mean (SD) number of appointments per patient over 1 year; Number of appointments (secondary outcome)  4a. Overall, high target INR  4b. Overall, low target INR  4c. Warfarin, high target INR  4d. Warfarin, low target INR  4e. Acenocoumarol, high target INR  4f. Acenocoumarol, low target INR  5. Mean (SD) dosage of anticoagulant drug (mg/week) over 1 year (secondary outcome).  5a. Warfarin, high target INR.  5b. Warfarin, low target INR.  5c. Acenocoumarol, high target INR.  5d. Acenocoumarol, low target INR.    6. Mean INR value over 1 year (secondary outcome).  6a. Overall, high target INR.  6b. Overall, low target INR.  6c. Warfarin, high target INR.  6d. Warfarin, low target INR.  6e. Acenocoumarol, high target INR.  6f. Acenocoumarol, low target INR.  Starting treatment group (enrolled before 2^nd^ visit and followed for ≥ 3 months, N=145 vs 190): 7**. Percentage of patients reaching stable condition (primary outcome). [Stable = 3 consecutive INRs within therapeutic range at least 1 week from each other].**  7a. 1-31 days.  7b. 1-60 days.  **7c. 1-90 days.**  7d. 1 to >90 days. 8. Percentage of time spent within the therapeutic range limit (secondary outcome).  8a. All months.  8ai. Overall (71.3 patient/years follow-up).  8aii. Warfarin patients (44.5 patient/years follow-up)  8aiii. Acenocoumarol patients (26.8 patient/years follow-up).  8b. 1^st^ month.  8bi. Overall.  8bii. Warfarin patients.  8biii. Acenocoumarol patients.  8c. 2^nd^ month.  8ci. Overall.  8cii. Warfarin patients.  8ciii. Acenocoumarol patients.  8d. 3^rd^ month.  8di. Overall.  8dii. Warfarin patients.  8diii. Acenocoumarol patients.  9. Percentage of time spent below the therapeutic range (secondary outcome)  9a. All months.  9ai. Overall (71.3 patient/years follow-up)  9aii. Warfarin patients (44.5 patient/years follow-up)  9aiii. Acenocoumarol patients (36.8 patient/years follow-up).  9b. 1^st^ month.  9bi. Overall.  9bii. Warfarin patients.  9biii. Acenocoumarol patients.  9c. 2^nd^ month.  9ci. Overall.  9cii. Warfarin patients.  9ciii. Acenocoumarol patients.  9d. 3^rd^ month.  9di. Overall.  9dii. Warfarin patients.  9diii. Acenocoumarol patients.  10. Percentage of time spent above the therapeutic range (secondary outcome).  10a. All months.  10ai. Overall (71.3 patient/years follow-up)  10aii. Warfarin patients (44.5 patient/years follow-up)  10aiii. Acenocoumarol patients (46.8 patient/years follow-up).  10b. 1^st^ month.  10bi. Overall.  10bii. Warfarin patients.  10biii. Acenocoumarol patients.  10c. 4^nd^ month.  10ci. Overall.  10cii. Warfarin patients.  10ciii. Acenocoumarol patients.  10d. 3^rd^ month.  10di. Overall.  10dii. Warfarin patients.  10diii. Acenocoumarol patients.  Note: Time spent in range for ‘starting treatment’ group also shown in figure 2. | Long term therapy group  N = 458 vs 458  1ai. 71.2% vs 68.2%, *P<*.001  1aii. 72.5% vs 70.5%, *P<*.001  1aiii. 68.7% vs 63.5%, *P<*.001  1bi. 70.6% vs 68.2%, *P<*.001  1bii. 73.9% vs 72.8%, *P<*.001  1biii. 64.6% vs 61.0%, *P<*.001  1ci. 71.6% vs 68.3%, *P<*.001  1cii. 71.7% vs 69.5%, *P<*.001  1ciii. 71.3% vs 65.3%, *P<*.001  2. p-values NR  2ai. 19.0% vs 21.4%  2aii. 17.5% vs 19.3%  2aiii. 22.0% vs 25.8%  2bi. 22.7% vs 25.5%  2bii. 19.4% vs 21.6%  2biii. 28.5% vs 31.8%  2ci. 17.0% vs 19.1%  2cii. 16.6% vs 18.1%  2ciii. 17.8% vs 21.5%  3. *P*-values NR  3ai. 9.8% vs 10.4%  3aii. 10.0% vs 10.2%  3aiii. 9.3% vs 10.7%  3bi. 6.7% vs 6.3%  3bii.6.7% vs 5.6%  3biii. 6.9% vs 7.2%  3ci. 11.4% vs 12.6%  3cii. 11.7% vs 12.4%  3ciii.10.9% vs 13.2%  4a. 18.6 (8.74) vs 19.5 (7.42), *P<*.001; 3,189 vs 3,257  4b. 15.7 (4.69) vs 16.8 (4.95), *P<*.001, 4,288 vs 4,505  4c. 18.4 (4.82) vs 19.4 (7.42), *P<*.001; 1,982 vs 1,995  4d. 15.6 (4.71) vs 16.3 (4.76), *P<*.001; 3, 192 vs 3,318  4e. 19.1 (9.82) vs 19.6 (5.04), NS; 1,207 vs 1,262  4f. 16.1 (4.63) vs 18.4 (4.82), *P<*.001; 1,106 vs 1,187  5a. 33.3 (15.7) vs 31.3 (12.8), *P<*.001  5b. 29.7 (12.9) vs 29.7 (14.4), NS  5c. 19.2 (9.82) vs 17.8 (10.4), *P<*.01  5d. 14.7 (6.70) vs 14.8 (6.81), NS  6a. 3.07 (1.01) vs 2.95 (0.84), *P<*.001  6b. 2.51 (0.82) vs 2.55 (0.76), NS  6c. 3.10 (0.93) vs 2.90 (0.69), *P<*.001  6d. 2.50 (0.76) vs 2.51 (0.75), NS  6e. 3.03 (1.05) vs 2.99 (0.99), NS  6f. 2.51 (0.85) vs 2.59 (0.81), NS  7a. 39% vs 27%, *P<*.01  7b. 73% vs 57%, *P<*.05  7c. 93% vs 87%, NS  7d. 100% vs 100%, NS  8ai. 51.9% vs 48.1%, *P<*.001  8aii. 52,2% vs 49.6%, *P<*.001  8aiii. 51.4% vs 45.3%, *P<*.001  8bi. 47.4% vs 44.0%, *P<*.001  8bii. 46.5% vs 45.4%, NS  8biii. 48.9% vs 41.1%, *P<*.001  8ci. 51.1% vs 45.2%, *P<*.001  8cii. 51.5% vs 47.3%, *P<*.01  8ciii. 50.5% vs 41.3%, *P<*.001  8di. 57.8% vs 56.4%, NS  8dii. 60.2% vs 57.7%, NS  8diii. 54.7% vs 54.2%, NS  9. *P*-values NR  9ai. 40.8% vs 43.3%  9aii. 41.6% vs 42.2%  9aiii. 39.6% vs 45.3%  9bi. 43.0% vs 43.2%  9bii. 45.8% vs 43.0%  9biii. 38.7% vs 43.8%  9ci. 42.8% vs 48.7%  9cii. 43.9% vs 47.2%  9ciii. 41.3% vs 51.6%  9di. 36.0% vs 37.0%  9dii. 33.9% vs 35.2%  9diii. 38.7% vs 40.1%  10. *P*-values NR  10ai. 7.3% vs 8.6%  10aii. 6.2% vs 8.2%  10aiii. 9,0% vs 9.4%  10bi. 9.6% vs 12.8%  10bii. 7.7% vs 11.6%  10biii. 12.4% vs 15.1%  10ci. 6.1% vs 6.1%  10cii. 4.6% vs 5.5%  10ciii. 8.2% vs 7.1%  10di. 6.2% vs 6.6%  10dii. 5.9% vs 7.1%  10diii. 6.6% vs 5.7% | … | … | + | … |
| Fitzmaurice, 2000 [25] | **1. Point prevalence of patients achieving therapeutic INR target over 12 months (primary outcome – 1 of 2). Baseline/Study % (95% CI) 2. Percentage of time spent in target INR range over 12 months (primary outcome – 1 of 2). Baseline/Study % (95% CI)**  3. Proportion of tests in INR range over 12 months. Baseline/Study % (95% CI)  Note: Target range varied by clinical indication for treatment: 2.0 to 3.0 or 3.0 to 4.5 | Intervention vs Interpractice control  1. 63% (54 to 71)/71% (63 to 79) vs 54% (46 to 62)/66% (58 to 73), NS  2. 57% (50 to 63)/69% (66 to 73) vs 62% (53 to 70)/65% (61 to 70), NS  3. 61% (55 to 67)/62% (58 to 66) vs 61% (53 to 68)/62% (58 to 66)) | **All prespecified (12 month study)**  **1. Serious adverse events.**  1a. Deep vein thrombosis.  1b. Transient Ischemic attack.  1c. Fatal cerebrovascular accident.  1d. Nonfatal cerebrovascular accident.  1e. Saddle embolus  1f. Epistaxis  **1g. Total**  **2. Cause of death.**  2a. Stroke.  2b. Congestive cardiac failure  2c. Ischemic heart disease.  2d. Left ventricular failure.  2e. Renal failure.  2f. Carcinoma.  **2g. Total.**  **3. Patient satisfaction.** | Intervention vs Interpractice control  Number of patients; patient-y follow-up per group = 87.3 vs 97.3  1a. 1//0  1b. 0/3/  1c. 1/1  1d. 0/3  1e. 0/0  1f. 1/0  1g. 3/7 (NS)  Number of patients; rand per group = 122/143  2a. 1/1  2b. 1/1  2c. 0/1  2d. 0/0  2e. 0/0  2f. 1/0  2g. 3/3 (NS)  3. Results not presented. | 0 | 0 |
| Ageno, 1998 [23] | Prespecified for 50 vs 51 patients and 14,419 vs 14,638 treatment days. **1. INRs within therapeutic range (2.5-3.5) over 10 months. 2. Dosage adjustments over 10 months, %; % difference. 3. Number of INR tests/number of patients over 10 months; mean number of tests; % difference.**  **4. Days within range (2.5-3.5) over 10 months.  5. Mean INR over 10 months.**  **6. % INRs >5.0 over 10 months. 7. % INRs <2.0 over 10 months.**  **8. Mean test interval (days) over 10 months; % difference. 9. Proportion interventions manually overridden over 10 months.**  Not prespecified: 10. INRs within satisfactory range (2.3-3.7) over 10 months. 11. Days within satisfactory range (2.3-3.7) over 10 months.  12. Number of changes ordered; % difference. | 1. 49.6% vs 51.5%, NS 2. 31.3% vs 47.4%; -34%, *P=*.02  3. 706/50 vs 866/51, 14.1 vs 16.9; -16.6% 4. 55.3% vs 55.2%, NS 5. 2.83 vs 3.12 6. 3% vs 2.9% 7. 10.2% vs 6.6% 8. 20.2 vs 17.0; 18.8% 9. 4.9%  10. 64.9% vs 64.6%, NS 11. 68.9% vs 68.8%, NS  12. 221 vs 410; -46.1% | … | … | … | … |
| Poller, 1998 [24] | 6-month study with ≥ 3 months follow-up  Data also reported by patient subgroups (below), study weeks (1-3, 4-9, 10-21, >22), and by each of 5 participating centres.  a) Stable on long-term anticoagulant therapy (most >22 wks therapy)  b) Stabilization group who were discharged from hospital within 6 wks of starting anticoagulation therapy.  **Prespecified: proportion of time in range.**  **1. Mean (SD) days within target INR range for all patients and all ranges (3 ranges used in study: 2-3, 2.5-3.5, and 3-4.5).**  **1a. All patients**  1b. Stabilization patients  1c. Stable patients  2. Total (122 vs 132 patients)  2a. Number of INRs.  **2b. Proportion of time in target range.**  2c. Mean days between visits.  2d. Proportion dose changes.  2e. Proportion low INRs.  2f. Proportion high INRs.  2g. Mean (SD) INR.  3. Stabilization patients – first 3 weeks  3a. Number of INRs.  3b. Proportion of time in target range.  3c. Mean days between visits).  3d. Proportion dose changes.  3e. Proportion low INRs.  3f. Proportion high INRs.  3g. Mean INR.  4. Stabilization patients (83 vs 92 patients) – weeks 4 to >22  4a. Number of INRs.  4b. Proportion of time in target range.  4c. Mean days between visits.  4d. Proportion dose changes.  4e. Proportion low INRs.  4f. Proportion high INRs.  4g. Mean (SD) INR.  5. Stable patients (39 vs 40 patients) – overall  5a. Number of INRs.  5b. Proportion of time in target range.  5c. Mean days between visits.  5d. Proportion dose changes.  5e. Proportion low INRs.  5f. Proportion high INRs.  5g. Mean (SD) INR.  6. Proportion low INRs  6a. Stabilization, INR target 2.0 to 3.0.  6b. Stabilization, INR target 2.5 to 3.5.  6c. Stabilization, INR target 3.0 to 4.5.  6d. Stable, INR target 2.0 to 3.0.  6e. Stable, INR target 2.5 to 3.5.  6f. Stable, INR target 3.0 to 4.5.  7. Proportion high INRs  7a. Stabilization, INR target 2.0 to 3.0.  7b. Stabilization, INR target 2.5 to 3.5.  7c. Stabilization, INR target 3.0 to 4.5.  7d. Stable, INR target 2.0 to 3.0.  7e. Stable, INR target 2.5 to 3.5.  7f. Stable, INR target 3.0 to 4.5.  8. Proportion time in INR ranges.  8a. Stable, All ranges  8b. Stable, INR target 2.0 to 3.0.  8c. Stable, INR target 2.5 to 3.5.  8d. Stable, INR target 3.0 to 4.5.  Note: data also reported for stabilization patients by INR target range but this is provided separately by weeks (4-9, 10-21, and >22), not overall. | 1a. 63.3% (28.0) vs 53.2% (27.7), *P=*.004  1b. 61.8% (27.1) vs 54.0% (27.5), *P=*.06  1c. 66.4% (29.9) vs 51.2% (28.4), *P=*.02  2a. 933 vs 1080  2b. 70% vs 56%  2c. 18 vs 17  2d. 38% vs 53%  2e. 28% vs 33%  2f. 15% vs 17%  2g. 2.6 (0.9) vs 2.6 (1.0)  3a. 40 vs 195  3b. 42% vs 45%  3c. 7 vs 7  3d. 55% vs 65%  3e. 28% vs 36%  3f. 38% vs 28%  3g. 3.0 vs 2.7  4a. 619 vs 693  4b. 68% vs 55%  4c. 17 vs 16  4d. 39% vs 57%  4e. 29% vs 36%  4f. 11% vs 16%  4g. 2.6 (0.8) vs 2.6 (1.1)  5a. 314 vs 387  5b. 72% vs 59%  5c. 20 vs 18  5d. 36% vs 46%  5e. 25% vs 27%  5f. 18% vs 19%  5g. 2.7 (0.9) vs 2.7 (0.8)  6a. 22.8% vs 32.2%  6b. 34.5% vs 44.3%  6c. 35.4% vs 44.7%  6d. 19.7% vs 23.0%  6e. 32.2% vs 23.3%  6f. 42.1% vs 46.4%  7a. 15.7% vs 17.7%  7b. 9.1% vs 19.7%  7c. 9.4% vs 10.5%  7d. 16.2% vs 19.4%  7e. 25.3% vs 18.3%  7f. 5.3% vs 7.1%  8a. 72.3% vs 59.3%  8b. 80.0% vs 59.9%  8c. 51.6% vs 72.5%  8d. 76.1% vs 46.3% | … | … | + | … |
| Vadher, 1997 [22] | Main outcomes **1. Median [SE] days to reach therapeutic range (INR ≥2).  2. Median [SE] days to reach stable dose (INR 2-3 for 3 consecutive days).  3. Median time to first pseudoevent (INR ≤1.5 or ≥5 after therapeutic range is reached).**  **For inpatient treatment (n=60 vs 62)**  **4. Days (per 100 patient days of treatment) at INR 2-3; relative rate (95% CI). (main outcome)**  **For outpatient treatment (n=53 vs 64) 5. Days (per 100 patient days of treatment) at INR 2-3; (relative rate (95% CI). (main outcome)**  Prespecified For inpatient treatment (n=60 vs 62) 6. Days (per 100 patient days of treatment) at INR <1.5; relative rate (95% CI). 7. Days (per 100 patient days of treatment) at INR <2.0; relative rate (95% CI). 8. Days (per 100 patient days of treatment) at INR >3.0; relative rate (95% CI). 9. Days (per 100 patient days of treatment) at INR >5.0; relative rate (95% CI).  For outpatient treatment (n=53 vs 64) 10. Days (per 100 patient days of treatment) at INR <1.5; relative rate (95% CI).  11. Days (per 100 patient days of treatment) at INR <2.0; relative rate (95% CI).  12. Days (per 100 patient days of treatment) at INR >3.0; relative rate (95% CI).  13. Days (per 100 patient days of treatment) at INR >5.0; relative rate (95% CI).  Not prespecified  14. Number of patients below therapeutic range at hospital discharge.  15. Number of patients who did not reach a stable dose before study endpoint.  16. Median {range} INR test interval in inpatients (days).  17. Median {range} INR test interval in outpatients (days). | RRs reported are inverse of those in the paper to be consistent with presentation of data as intervention vs control.  1. 3 [0.34] vs 3 [0.29], *P=*.24  2. 7 [0.43] vs 9 [1.8], *P=*.01  3. Rates not reported, *P=*.06  4. 59.4 vs 52.2; 1.11 (1 to 1.43)  5. 63.7 vs 51.0; 1.25 (1.11 to 1.42)  6. 1.3 vs 5.6; 0.24 (0.13 to 0.45)  7. 18.3 vs 21.4; 0.83 (0.59 to 1.25)  8. 22.3 vs 26.4; 0.83 (0.59 to 1.25).  9. 1.2 vs 2.8; 0.42 (0.10 to 1.67)  10. 1.3 vs 4.2; 0.30 (0.11 to 0.77)  11. 21.1 vs 31.8; 0.67 (0.48 to 0.91)  12. 15.1 vs 17.2; 0.91 (0.56 to 1.43)  13. 0.8 vs 1.1; 0.67 (0.07 to 5)  14. 4/72 vs 8/76  15. 11/72 vs 14/76  16. 2 {1 to 22} vs 2 {1 to 30}, *P=*.07  17. 14 {2 to 63} vs 14 {1 to 91}, *P=*.2 | **Prespecified with median follow-up of 88-93 days. 1. Number of deaths.  2. Number of patients with haemorrhage events.  3. Number of patients with thromboembolism events.** | 1. 2/72 vs 2/76  2. 2/72 vs 4/76  3. 4/72 vs 1/76 | 0 | … |
| Fitzmaurice, 1996 [20] | **1. INR control (not defined) at 12 months (prespecified)**  2. Recall time over 12 months (not clearly prespecified) | 1. Control patients consistently undercoagulated compared with intervention patients but comparative data not provided.  2. Mean recall times not provided, but reported as increased for intervention vs control groups. | **All prespecified (12-month study)**  **1a. Deaths.**  **1b. Thrombotic episodes.**  **1c. Hemorrhagic episodes.**  **2. Patient satisfaction.** | No analyses done.  1a. 1/14 vs 2/9  1b. 1/14 vs 0/9  1c. 3 (2 epistaxis, 1 bruising) vs 5 (3 epistaxis, 1 gum bleed, 1 hematoma); Author reported that each event was for 1 patient only.  2. Data not provided by group; no analysis | … | … |
| Fihn, 1994 [19] | Mean patient follow-up: 8 months. **1. Ability to increase intervals between visits for CCDSS (n=301 patients) vs control (n=319 patients): mean (SD) number of weeks.**  1a. Recommended return interval. **1b. Scheduled return interval (primary). 1c. Actual return interval (primary).   2. Mean (SD) absolute deviation of measured prothrombin times (PTs) and INRs from their target values (primary). 2a. PT. 2b. INR.**  Secondary outcome 3. Frequency of dosage changes (dose changes per year). | 1a. 5.5 (2.1) vs 5.2 (2.2), NS  1b. 4.4 (1.8) vs 3.5 (1.4), *P<*.001  1c. 4.4 (1.8) vs 4.1 (1.8), *P<*.05  2a. 0.19 (0.16) vs 0.18 (0.09), NS  2b. 0.71 (1.21) vs 0.66 (0.40), NS  3. 11.2 vs 11.8 | **Prespecified outcomes; mean follow-up 8 months (N=301 vs 319). 1. Clinically important bleeding: Number of patients; incidence per 100 patient years.** 1a. Serious events. 1b. Life-threatening events. **1c. Relative risk (95% CI) for bleeding complications adjusted for anticoagulation intensity.**  **2. Thromboembolic complications: Number of patients; incidence per 100 patient-years.** 2a. Serious events. 2b. Life-threatening events.  **2c. Relative risk (95% CI) for thromboembolic complications adjusted for anticoagulation intensity. 3. Deaths.**  Not prespecified  4. Proportion of hemorrhagic complications that occurred when prothrombin ratio > 2.0.  5. Number of patients with a 2^nd^ complication. | 1a. 11 vs 14; 5.4 vs 6.7  1b. 2 vs 1; 1.0 vs 0.5, *P=*.74 for 1a and 1b combined.  1c. 1.1 (0.5 to 2.3)  2a. 5 vs 3; 2.4 vs 1.4  2b. 1 vs 0; 0.5 vs 0, *P=*.28 for 2a and 2b combined.  2c. 2.1 (0.5 to 8.4)  3. No deaths occurred.  4. 15% vs ~50%  5. 3 vs 3 | + | 0 |
| Poller, 1993 [18] | Follow-up period unclear  **1. Number, proportion, of visits spent in or out of target range (“INR” was prespecified) 1a. All patients - in range.**  1b. All patients - below range.  1c. All patients - above range.  1d. New patients (n=116) – in range.  1e. New patients – below range.  1f. New patients – above range.  1g. Long-term warfarin patients (n=58) – in range.  1h. Long-term warfarin patients – below range.  1i. Long-term warfarin patients – above range.  2. Percentage of visits within or outside of range for INR target range 2.0 to 3.0 (“INR” was prespecified) 2a. In range.  2b. Below range.  2c. Above range.  3. Percentage of visits within or outside of range for INR target range 3.0 to 4.5 (“INR” was prespecified) 3a. In range.  3b. Below range.  3c. Above range **4. Mean time between visits (in weeks) (suggested interval was prespecified)**  **4a. Overall.**  4b. For 116 new patients.  4c. For 58 patients on long-term warfarin.  5. Percentage of visits in different INR ranges.  5a. INR <2.0.  5b. INR 2.0 to 4.0.  5c. INR 2.0 to 4.5.  5d. INR >4.5.  Note: Hillingdon system was discontinued during the study and is not included in this review. | Charles vs Coventry vs Usual dosing  1a. 96/170, 56.5%, vs 68/128, 53.1%, vs 118/234, 50.4%, NS  1b. 47/170, 27.6%, vs 32/128, 25%, vs 75/234, 32.1%  1c. 27/170, 15.9%, vs 28/128, 21.9%, vs 41/234, 17.5%  1d. 55.7% vs 54.3% vs 50.8%  1e. 29.0% vs 29.6% vs 35.9%  1f. 15.3% vs 16.0% vs 13.3%  1g. 59.0% vs 51.1% vs 49.1%  1h. 23.1% vs 17.0% vs 18.9%  1i. 17.9% vs 31.9% vs 32.1%  2a. 56.8% vs 51.5% vs 59.7%, *P=*.62  2b. 27.4% vs 28.3% vs 20.9%  2c. 15.8% vs 20.2% vs 19.4%  3a. 56.0% vs 58.6% vs 36.8% (Charles and Coventry significantly diff from traditional dosing, *P=*.04)  3b. 28.0% vs 13.8% vs 48.4%  3c. 16.0% vs 27.6% vs 14.7%  4a. 2.9 vs 3.2 vs 3.1, NS  4b. 2.6 vs 3.1 vs 2.6  4c. 3.9 vs 3.4 vs 4.9  5a. 15.3% vs 22.7% vs 17.7%  5b. 68.2% vs 68.8% vs 69.6%  5c. 75.9% vs 71.7% vs 74.0%  5d. 8.8% vs 6.3% vs 8.1% | **All prespecified (follow-up period unclear)**  **1. Major bleeding events. 2. Other clinical events. 3. Deaths.** | No analyses done.  1. 0/57 vs 0/53 vs 0/64  2. 0/57 vs 0/53 vs 0/64  3. 0/57 vs 1/53 vs 0/64 | 0 | 0 |
| White, 1991 [15] | **Not clearly prespecified**  **1. Mean (SD) absolute difference between achieved and target PTs at median 14 day follow-up (seconds); 95% CI for difference.**  **2. Mean (SD) % difference between achieved and target PTs at median 14 day follow-up.  3. Number, proportion, of patients with final PT within 2 seconds of target at median 14 day follow-up.  4. Mean (SD) % change in warfarin dose at median 14 day follow-up.**  **5. Mean (SD); Median {range} follow-up interval (days).** | 1. 2.3 (1.37) vs 2.6 (2.20); -1.0 to 1.6, NS  2. 14% (10) vs 13% (10), NS  3. 10/23, 43% vs 12/24, 50%, NS  4. 20% (17) vs 15% (11), NS  5. 18.7 (13) vs 17.5 (10); 14 {7 to 42} vs 14 {7 to 37}, NS | … | … | 0 | … |
| Carter, 1987 [9] | **1. For patients who achieved a stable PT ratio before discharge, mean (SD) number of days from administration of first warfarin dose to achievement of stabilization dosage (prespecified).**  2. Proportion of patients with stable PT before or at hospital discharge (not prespecified).  3. Mean (SD) stabilization warfarin dosage (mg/day) (not prespecified).  4. Proportion of PT ratios within each PT ratio category as measured between the time of the third warfarin dose and achievement of either a stable PT ratio or discharge (not prespecified).  4a. PT ratio ≤1.3  4b. PT ratio 1.31 to 2.0  4c. PT ratio 2.01 to 2.5  4d. PT ratio ≥2.5  Actual versus predicted dosages for various warfarin dose numbers in analog group also provided | Analog vs Linear vs Empiric groups  1. 6.8 (1.26) vs 7.33 (2.06) vs 8.42 (3.47), NS  2. 20/31, 64.5% vs 15/22, 68.2% vs 19/34, 55.9%  3. 7.16 (4.41) vs 7.44 (2.6) vs 7.82 (3.2)  4a. 2.4% vs 9.6% vs 13.1%  4b. 88.3% vs 63.8% vs 81.7%  4c. 6.7% vs 24.5% vs 5.2%  4d. 0.8% vs 2.1% vs 0%  No statistical analyses provided for these measures. | … | … | 0 | … |
| White, 1987 [10] | **Prespecified 1. Mean (not clear if SD or SE) time to reach a stable therapeutic dose (days).  2. Mean time to reach a therapeutic PR ratio (days).  3. Number of patients with PR above therapeutic range during hospital stay.  4. Mean predicted/observed PR.  5. Mean absolute error (absolute value of absolute PR – predicted PR).**  Not prespecified 6. % mean absolute error.  7. Mean days on warfarin with PR in therapeutic range during hospital stay.  8. Mean days on warfarin with PR above therapeutic range during hospital stay.  9. Mean days on warfarin with PR below therapeutic range during hospital stay.  10. Number of patients reaching PR therapeutic range after 6 days.  11. Number of patients reaching a stable therapeutic dose after 10 days. 12. Mean warfarin dose at discharge (mg/day).  13. Proportion of patients with PR in therapeutic range 10-14 days after start of maintenance dose.  14. Number of patients with PR above therapeutic range10-14 days after start of maintenance dose.  15. Number of patients with PR below therapeutic range10-14 days after start of maintenance dose  16. Mean time on warfarin (days),  17. Number (proportion) of patients discharged on warfarin <5.0 mg/day. | 1. 5.7 (1.7) vs 9.4 (5.2), *P=*.002  2. 3.2 (1.6) vs 4.5 (3.4), *P=*.05 (Note 1)  3. 2/39 vs 6/36, *P=*.11  4. 1.75 (0.2)/ 1.76 (0.3) vs 1.67 (0.1)/ 1.94 (0.9), NS  5. 0.20 (0.2) vs 0.62 (0.7), *P=*.05 (Note 1)  6. 13% (14) vs 30% (19), *P=*.05 (Note 1)  7. 58% (23) vs 42% (27), *P=*.001  8. 3.0% (9) vs 5.9% (14), NS  9. 39% (24) vs 51% (31), NS  10. 1/39 vs 6/36  11. 0/39 vs 11/36  12. 5.9 vs 7.1  13. 28/33, 85%, vs 11/26, 42%, *P=*.002  14. 2/33 vs 8/26 [Note: text and table data reversed for this outcome]  15. 3/33 vs 7/26 [Note: text and table data reversed for this outcome]  16. 8.9 (6.8) vs 11.3 (8), NS  17. 12, 36%, vs 4, 15%  Note 1: Author indicates *P<*.05 as significant but reports this comparison as significant. Unable to confirm with author. | **Prespecified**  **1. Mean (not clear if SD or SE) length of hospital stay (days).**  **2. Number patients with in-hospital bleeding complications (major/minor) during hospital stay.**  Not prespecified.  3. Number of deaths.  4. Number of patients with thromboembolic complications on warfarin therapy. | 1. 13 (8) vs 20 (15), *P=*.01  2. 0/39 vs 1(2)/36, NS  3. 0/39 vs 0/36  4. 0/33 vs 0/26 | + | + |
| **Aminophylline and Theophylline Dosing** | | | | | | |
| Tierney, 2005 [31] | Physician intervention vs pharmacist intervention vs both interventions vs control. Number of patients/group, 194 vs 161 vs 182 vs 169 Primary outcome **1. Number of suggestions adhered to/Number of patients with suggestions; % of care suggestions adhered to over 3 yrs.** 1a. Overall.  **1b. Increase/decrease theophylline dose.**  **Prespecified**  **2. Medication compliance measures.  2a. Mean Inui score (%).  2b. Mean (SD) Morisky score.  2c. Proportion of patients with ≥2 prescription refills.  2d. Mean (SD) medication possession ratio (measure referenced but not described).  3. Mean (SD) score for patient satisfaction with physician (American Board of Internal Medicine questionnaire, score range/direction not described).  4. Mean (SD) score for patient satisfaction with pharmacist (American Board of Internal Medicine questionnaire, score range/direction not described).** | 1a. 161/498, 32% vs 123/382, 32% vs 173/471, 37% vs 135/416, 32%, NS  1b. 26/39, 67% vs 18/25, 72% vs 20/31, 65% vs 16/24, 67%, NS  All NS  2a. 81% vs 80% vs 82% vs 80%  2b. 0.95 (1.1) vs 0.85 (1.0) vs 0.89 (1.1) vs 0.88 (1.0)  2c. 95% vs 81% vs 92% vs 87%  2d. 0.98 (0.8) vs 1.00 (2.7) vs 1.1 (2.0) vs 0.92 (1.0)  3. 1.9 (0.9) vs 2.0 (0.9) vs 2.1 (0.6) vs 2.1 (0.7), NS  4. 2.1 (0.7) vs 2.1 (0.8) 2.0 (0.6) vs 2.1 (0.7), NS | Physician intervention vs pharmacist intervention vs both interventions vs control **All Prespecified with follow-up at 12 months. 1. Mean (SD) short-form 36 subscale scores (N/group: 135 vs 110 vs 118 vs 111). Higher scores better.  1a. Physical function.  1b. Role physical.  1c. Pain.  1d. General health.  1e. Vitality.  1f. Social function.  1g. Role emotional.  1h. Mental health.   2. Mean (SD) McMaster Asthma Quality of Life Questionnaire subscale scores (N/group: 38 vs 31 vs 27 vs 20). Higher scores better.  2a. Overall health status.**  2b. Activity.  2c. Symptoms.  2d. Emotion.  2e. Environment.   **3. Mean (SD) McMaster Chronic Respiratory Disease Questionnaire subscale scores (N/group: 72 vs 104 vs 91 vs 91). Higher scores better.  3a. Overall health status.**  3b. Dyspnoea.  3c. Fatigue.  3d. Emotion.  3e. Mastery.   **5. Mean (SD) number of emergency department visits.  5a. For any reason.**  5b. For reactive airways disease.  **6. Mean (SD) number of hospitalizations. 6a. For any reason.**  6b. For reactive airways disease. | All NS unless noted otherwise.  1a. 38 (23) vs 38 (27) vs 36 (24) vs 37 (26)  1b. 32 (40) vs 33 (40) vs 38 (41) vs 32 (40), *P<*.05 in favour of both interventions  1c. 49 (25) vs 47 (27) 48 (26) vs 44 (26)  1d. 37 (24) vs 29 (25) vs 35 (20) vs 34 (22)  1e. 37 (21) vs 39 (23) vs 36 (23) vs 36 (20)  1f. 69 (27) vs 63 (30) vs 61 (29) vs 63 (29)  1g. 65 (43) vs 60 (44) vs 59 (43) vs 60 (45)  1h. 62 (23) vs 62 (23) vs 50 (25) vs 61 (24)  2a. 4.0 (1.5) vs 4.2 (1.4) vs 4.2 (1.1) vs 3.7 (1.3)  2b. 4.5 (1.5) vs 4.6 (1.3) vs 4.4 (1.2) vs 3.9 (1.2)  2c. 4.0 (1.5) vs 4.0 (1.5) vs 4.2 (1.2) vs 3.6 (1.4)  2d. 3.8 (2.0) vs 4.3 (1.6) vs 4.4 (1.2) vs 3.6 (1.5), *P<*.05 in favour of pharmacist intervention  2e. 3.9 (1.6) vs 4.2 (1.5) vs 4.0 (1.4) vs 3.7 (1.4)  3a. 4.4 (1.2) vs 4.3 (1.3) vs 4.1 (1.1) vs 4.2 (1.1)  3b. 4.2 (1.6) vs 4.2 (1.7) vs 4.0 (1.6) vs 4.0 (1.5)  3c. 3.8 (1.3) vs 3.7 (1.5) vs 3.4 (1.2) vs 3.6 (1.2)  3d. 4.6 (1.3) vs 4.5 (1.4) vs 4.2 (1.2) vs 4.4 (1.3)  3e. 4.8 (1.4) vs 4.8 (1.5) vs 4.5 (1.4) vs 4.6 (1.4)  5a. 1.4 (1.7) vs 1.5 (2.3) vs 1.4 (2.1) vs 1.4 (1.9)  5b. 0.3 (0.7) vs 0.4 (0.8) vs 0.4 (0.8) vs 0.3 (0.8)  6a. 0.5 (1.6) vs 0.5 (1.1) vs 0.4 (1.1) vs 0.4 (0.8)  6b. 0.1 (0.5) vs 0.1 (0.5) vs 0.1 (0.5) vs 0.1 (0.3) | 0 | 0 |
| Casner, 1993 [17] | **Prespecified (time NR). 1. Mean (SD) serum theophylline levels (mg/L).**  **1a. ≥ 8 hours after intravenous therapy had been initiated (C1).**  **1b. ≥ 6 hours after the first measurement (C2).**  **1c. Just before discontinuation of the intravenous theophylline infusion (C3).**  **1d. Time interval (mean or median not specified) between C1 and C3 (hours).**  **2. Mean (SD) absolute difference between final and target (15 mg/L) theophylline levels (mg/L).  3. Mean (SD) difference between target (15 mg/L) and mean final theophylline level (mg/L).  4. Number of patients with subtherapeutic (<10 mg/L) final theophylline levels.  5. Number of patients with toxic (>20 mg/L) final theophylline levels.**  Not clearly prespecified  6. Mean (SD) number of days of theophylline administration.  7. Mean (SD) prediction error. | 1a. 10.2 (6.4) vs 9.8 (3.9), NS  1b. 10.6 (3.3) vs 9.7 (3.2), NS  1c. 14.8 (4.4) vs 12.6 (4.1), NS  1d. 48 vs 40, NS  2. 3.5 (2.7) vs 3.9 (2.6), NS  3. 0.21 (4.49) vs 2.41 (4.07), NS  4. 4 vs 3, NS  5. 1 vs 1, NS  6. 4.1 (3.3) vs 3.2 (1.5), NS  7. 0.21 (4.49) vs 2.41 (4.07), NS | **Not clearly prespecified. 1. Number of patients with theophylline-associated toxicity (nausea, vomiting, tremor, tachycardia, and seizures) (follow-up time NR). 2. Mean (SD) length of hospital stay (days).**  2a. Mean length of hospitalization without one outlier in each group (days). **3. Mean (SD) duration of treatment (days).** | 1. 1/17 vs 0/18. Event was tachycardia secondary to high initial theophylline level.  2. 11.4 (21.6) vs 8.8 (15.4), NS  2a. 6.1 vs 5.2, NS  3. 4.1 (3.3) vs 3.2 (1.5), NS | 0 | 0 |
| Gonzalez, 1989 [12] | Outcomes not clearly prespecified. 1**. Mean (SD) theophylline level (mg/L);** baseline, 6.7 (5.2) vs 6.8 (6.0), NS. **1a. 1h.  1b. 2h.  1c. 4h.** | 1a. 14.0 (2.5) vs 12.5 (3.7), NS  1b. 14.6 (2.7) vs 12.2 (3.8), *P<*.002  1c. 14.6 (3.1) vs 11.4 (3.9), *P<*.001 | **Outcomes not clearly prespecified; 82 randomized; 37 vs 30 analyzed**  **1. Patients discharged from emergency department within 8 hours (i.e., not admitted to hospital).  2. Proportion of patients with adverse effects (nausea and vomiting) in emergency department.**  **3. Peak flow rate throughout the study.** | (Number of patients NR, only %).  1. 52% vs 47%, *P<*.7  2. 10% vs 7%, *P<*.7  3. Values not given, did not differ | + | 0 |
| Hurley, 1986 [8] | **Prespecified 1. Patients with theophylline levels above therapeutic range (10-20 µg/mL) on days 1 and 2.  2. Patients with theophylline levels below therapeutic range (10-20 µg/mL) on days 1 and 2. 3. Patients with trough theophylline levels in therapeutic range during oral therapy.  4. Mean (SD) serum theophylline levels (µg/mL) Day 1**  **Day 2**  **5. Mean (SD) 1st serum level during oral therapy (µg/mL): 6. Mean (SD) trough levels during oral therapy (µg/mL).**  Not prespecified 7. Mean (SD) IV aminophylline infusion rate (mg/kg IBW/h) Day 1  Day 2  8. Mean (SD) IV aminophylline infusion duration (hours)  9. Mean (SD) hydrocortisone dose, day 1  10. Number (proportion) of patients given hydrocortisone + prednisolone during admission. | 1. day 1 NS  day 2 18.9% vs 37.8%, *P=*.04  2. day 1 3/47 vs 4/41, *P=NS (*Yates-corrected Chi² = 0.035519 P = .8505*, calculated by Research Associate)*  day 2 4/37 vs 1/37 *P=NS (*Yates-corrected Chi² = 0.857971 P = .3543, calculated by Research Associate*)*  3. 71.1% vs 44.4%, *P=*.02  4.  day 1 14.9 (3.5) vs 15.8 (6.1), NS overall, *P<*.01 for variance  day 2 16.1 (5.2) vs 17.9 (7.0), NS overall, *P<*.05 for variance.  5. 12.9 (4.7) vs 10.8 (4.6), *P=*.03  6. 12.6 (3.9) vs 9.9 (4.1), *P=*.009  7.  day 1. 0.70 (0.21) vs 0.68 (0.15), NS overall, *P<*.05 for variance  day 2. 0.78 (0.33) vs 0.67 (0.19), NS, *P<*.01 for variance  8.  day 1. 24.0 (3.0) vs 22.8 (4.4), NS overall, *P<*.05 for variance  day 2. 22.4 (5.4) vs 22.1 (5.5), NS overall, *P<*.01 for variance  9. 725 (339) vs 792 (292), NS  10. 36/48, 75% vs 33/43, 76.7% | N = 48 vs 43; Other than death, number of patients NR for outcomes 2 and 3, only %.  **Prespecified 1. Mean daily peak expiratory flow rate (day 1, 2, and 3).**  **2. Patients with air flow obstruction symptoms during hospitalization; data not reported for all days.**  **2a. Severe breathlessness (%, d2 and d3).**  **2b. Wheeziness (not reported by day).**  **2c. Night wheeze (not reported by day).**  **2d. Cough (not reported by day).**  **3. Patients with side effects during hospitalization; data not reported for all days.**  **3a. Severe palpitations, d2 & d3.**  **3b. Nausea (not reported by day).**  **3c. Tremulousness (not reported by day). 3d. Agitation (not reported by day).**  **3e. Blurred vision (not reported by day).**  **3f. Diarrhoea (not reported by day).**  **3g. Deaths (n) during 6.3-8.7 days hospitalization.**  Not prespecified 4. Mean (SD) days in hospital. | 1. Higher for intervention patients (data shown only in figure), day 1 *P=*.07; day 2 *P=*.01, day 3 *P=*.09  2a. d2 31% vs 48.7%, *P=*.04; d3 16.6% vs 50%, *P=*.01 2b. NS (no data reported).  2c. NS (no data reported).  2d. NS (no data reported).  3a. d2 31% vs 66.7%, *P=*.003; d3 16.6% vs 56.2%, *P=*.001 3b. NS (no data reported).  3c. NS (no data reported).  3d. NS (no data reported).  3e. NS (no data reported).  3f. NS (no data reported). 3g.c. 0 vs 2  4. 6.3 (4.5) vs 8.7 (6.7), *P=*.03 | 0 | 0 |
| **Insulin Dosing and Glucose / Glycaemic Regulation** | | | | | | |
| Cavalcanti, 2009 [39] | All outcomes are presented in the order: **CCDSS vs** Leuven vs **Conventional.**  **1. Median (IQR) number of blood glucose measurements obtained per patient (secondary).**  **2. Mean (SD) proportion of time with blood glucose controlled between 60 and 140 mg/dL (secondary).** | 1. 100 (33 to 192) vs 105 (35 to 312) vs 49 (39 to 77);  *P=*.52 [CCDSS vs Leuven]; *P=*.01 [CCDSS vs Conventional]  2. 71.8 (18.0) vs 67.9 (20.8) vs 47.1 (30.2);  *P=*.50 [CCDSS vs Leuven]; *P*<.001 [CCDSS vs Conventional] | All outcomes are presented in the order: **CCDSS vs** Leuven vs **Conventional.**  **1. Mean of patients’ median blood glucose during the stay in intensive care unit (mg/dL) (primary).**  **2. Number, proportion, of patients with hypoglycaemia (≥ 1 blood glucose measurement ≤ 40 mg/dL) (primary).**  3. Mean of proportion of patients’ glucose measurements ≤40 mg/dL (secondary) (inconsistency < or ≤40 mg/dL).  4. Median (IQR) hyperglycaemic index, with a cutoff at 140 mg/dL (mg/dL per hour) (secondary). | 1. 125.0 vs 127.1 vs 158.5;  *P=*.34 [CCDSS vs Leuven]; *P<*.001 [CCDSS vs Conventional].  2. 12, 21.4% vs 24, 41.4% vs 2, 3.8%;  *P=*.02 [CCDSS vs Leuven]; *P=*.006 [CCDSS vs Conventional]  3. 0.43 vs 0.55 vs 0.03;  *P=*.04 [CCDSS vs Leuven]; *P=*.007 [CCDSS vs Conventional]  4. 4.2 (2.0 to 9.6) vs 8.7 (2.5 to 20.2) vs 20.5 (5.1 to 42.8);  *P=*.10 [CCDSS vs Leuven]; *P<*.001 [CCDSS vs Conventional] | + | + / − |
| Saager, 2008 [38] | **Primary outcome=decrease in blood glucose.**  **1 Operating room outcomes:**  **1a. Blood glucose in range 90 to 150 mg/dL (%).**  **1b. Time in range 90 to 150 mg/dL (minutes)(?mean, SD).**  **2. Intensive care unit outcomes:**  **2a. Blood glucose in range 90 to 150 mg/dL (%).**  **2b. Time in range 90 to 150 mg/dL (minutes)(?mean, SD).** | 1a. 49% vs 27%, *P<*.001  1b. 121 (67) vs 64 (85), *P=*.02  2a. 84% vs 60%, *P<*.001  2b. 536 (135) vs 377 (214), *P=*.01 | **Primary outcome=decrease in blood glucose.**  **1 Operating room outcomes:**  **1a. Mean (?SD) blood glucose (mg/dL).**  **1b. Mean (?SD) time to blood glucose<150 mg/dL (min).**  **2. Intensive care unit outcomes:**  **2a. Mean (?SD) blood glucose (mg/dL).**  **2b. Mean (?SD) time to blood glucose<150 mg/dL (min).**  (Outcomes not prespecified)  3. Number of episodes of hypoglycaemia (blood glucose <60 mg/dL).  3a. Operating room.  3b. Intensive care unit:  4. Median intensive care unit length of stay, days (IQR)  5. Hospital length of stay, days (IQR)  6. Postoperative complications (arrhythmias, prolonged intubation, infection, stroke or myocardial infarction). | 1a. 147 (19) vs 177 (36), *P<*.001  1b. 62 (92) vs 91 (121), *P=*.55  2a. 126 (18) vs 147 (27), *P=*.01  2b. 40 (97) vs 171 (238), *P=*.02  3a. 1 vs 0; NS  3b. 4 vs 1; *P=*.60  Note: 3 of 4 episodes of hypoglycaemia in the intensive care unit occurred within the same patient.  4. 2.5 (2 to 6) vs 2.5 (2 to 4.75); *P=*.83  5. 9.5 (6 to 11.75) vs 7.0 (6 to 11.75); *P=*.18  6. No differences; data not reported. | + | + |
| Albisser, 2007 [33] | **Secondary.**  **1. Mean (SD) daily insulin (U/day) over 2 months.** Not specified 2. Mean (SD) number of days to change hypoglycaemia episodes/wk (corresponds with patient outcome #1). 3. Range for dosing adjustments range over 2 months (U/day). | 1. 37 (16) vs 43 (16), *P<*.01  2. 46 (16) vs 61, NS  3. -27 to 0 vs -4 to 16, NS | **Primary 1. Mean (SD) hypoglycaemia episodes/week over 2 months.** Secondary 2. Mean (SD) glycated haemoglobin A1c over 2 months. 3. Pre-meal glycaemia shown for each group in figure 3 of article. | 1. 0.2 (0.3) vs 2.0 (0.9), *P<*.001. (N rand = 11 vs 11; in study group, most <=1 episode/month).  2. 7.5% (0.9) vs 7.6% (1.3), p = NS  3. No data reported. | + | + |
| Rood, 2005 [30] | **1. Deviation between advised and actual glucose measurement times over 10 weeks; (prespecified); Total number of samples 2352 vs 2597**  **1a. For late measurements: Mean minutes (SD); proportion of time (SD); difference in proportion of time (95% CI).**  **1b. For early measurements: proportion of time (SD); difference in proportion of time (95% CI).**  **2. Proportion of time that patients’ glucose levels were within specified range over 10 weeks; observed difference (95% CI).**  **2a. Target range, 4.0 to 7.0 mmol/L (prespecified).**  2b. <2.5 mmol/L (not clearly prespecified).  2c. 2.5 to 4 mmol/L (not clearly prespecified).  2d. 7 to 8.5 mmol/L (not clearly prespecified).  2e. >8.5 mmol/L (not clearly prespecified).  3. Proportion of dosing recommendations followed over 10 weeks; observed difference (95% CI) (not prespecified).  **4. % adherence to guideline for timing of glucose measurement over 10 weeks; observed difference (95% CI).**  **4a. % samples taken on time (prespecified).**  4b. % samples taken too late (not clearly prespecified).  4c. % samples taken too early (not clearly prespecified). | 1a. 27.95 (118.3) vs 42.49 (139.5); 28.1% (103.3) vs 41.9% (99.1); 14% (11 to 16)  1b. 27.8% (28.8) vs 28.95% (29.3)  2a. 54.2% vs 52.9%; 1.3% (1.0 to 1.56)  2b. 0.09% vs 0.05% (diff NR)  2c. 1.28% vs 1.32% (diff NR)  2d. 26.64% vs 27.53% (diff NR)  2e. 17.79% vs 18.21% (diff NR)  3. 77.3% vs 64.2%; 13.1% (11 to 16): total N of samples: 2352 vs 2597  4. total N of samples: 2352 vs 2597  4a. 40.18% vs 35.54%; 4.6% (2.0 to 7.4)  4b. 25.51% vs 31%; 5.5% (3.0 to 8.0)  4c. 34.31% vs 33.46% (difference NR) | … | … | + | … |
| Ryff-de Léche, 1992 [16] | **Not prespecified**  **1. Mean [SE] proportion of blood glucose levels in low range (< 4.0 mmol/L) from baseline to crossover (first 3 months); Baseline/Crossover. 2. Mean [SE] proportion of hypoglycaemic blood glucose levels < 2.9 mmol/L from baseline to crossover (first 3 months); Baseline/Crossover. 3. Mean [SE] proportion of blood glucose levels in target range (4.0 to 10.0 mmol/L) from baseline to crossover (first 3 months); Baseline/Crossover.** | 1. 12.4 [2.0]/10.2 [1.7] vs 15.3 [3.5]/13.0 [3.2]; p < 0.005 for change from baseline for each group; *P*=NR for comparison across groups. 2. 5.1 [1.1]/3.9 [0.9] vs 6.9 [1.7]/2.6 [0.7]; NS for change from baseline for each group; *P*=NR for comparison across groups. 3. 50.8 [4.1]/49.3 [4.9] vs 52.6 [4.1]/54.6 [5.8]; NS for change from baseline for each group; *P*=NR for comparison across groups | **Prespecified**  **1. Mean [SE] haemoglobin A1c values from baseline to crossover (first 3 months); Baseline/Crossover.** | 1. 6.8% [0.3]/6.3% [0.3] vs 7.0% [0.2] /6.7% [0.3], *P*<.005 for change from baseline in each group; *P*=NR for comparison between groups. | … | … |
| McDonald, 1976 [5] | **1. Number, proportion, of events to which provider responded by ordering the required tests to monitor drug effects over 8 months (prespecified).**  **1a. Overall.**  **2. Number, proportion, of events (abnormal measures) to which provider responded by changing therapy appropriately over 8 months (prespecified).**  **2a. Overall.**  3. Number, proportion, of events (abnormal measures) to which provider responded by changing therapy appropriately or repeating index measure over 8 months (not clearly prespecified). | 1a. 144/390, 36% vs 45/402, 11%, *P<*.001  2a. 31/110, 28% vs 9/68, 13%, *P<*.03  3. 63/110, 57% vs 16/68, 23%, *P<*.001 | … | … | + | … |
| **Aminoglycoside Dosing** | | | | | | |
| Burton, 1991 [14] | **Not clearly prespecified (follow-up unclear). 1. Number, proportion, of patients with peak aminoglycoside level > 4mg/L.  2. Number, proportion, of patients with trough aminoglycoside levels ≥2mg/L.** | 1. 58/70, 82.9% vs 44/73, 60.3%, NS  2. 6/69, 8.7% vs 11/75, 14.7%, NS | **Prespecified (follow-up unclear) 1. Proportion of patients cured.  2. Proportion of patients with response to therapy.  3. Proportion of patients with treatment failure.  4. Proportion of deaths.  5. Proportion of patients with indeterminate response.  6. Proportion of patients with nephrotoxicity.**  **7. Mean [SE] length of hospital stay (days).  8. Mean length of hospital stay after start of antibiotics (days).**  8a. Sepsis.  8b. Pneumonia.  8c. Cellulitis.  8d. Soft-tissue infections.  8e. Urinary tract infection.  8f. Gangrene.  8g. Postoperative wound infection.  8h. Peritonitis.  8i. Neutropenic, empiric therapy.  8j. Osteomyelitis.  8k. Cholangitis/cholecystitis.  8l. Catheter-tip infection.  8m. Subacute bacterial endocarditis.  8n. Septic arthritis.  8o. Pyelonephritis.  **8p. Overall**  **9. Mean [SE] length of aminoglycoside therapy (days).** | 1. 25.7% vs 25.3%. NS  2. 60% vs 48%, NS  3. 2.9% vs 5.3%, NS  4. 1.4% vs 4%, NS  5. 7.1% vs 8%, NS  6. 5.6% vs 9.3%, NS (calculated by Research Assistant for 4/72 vs 7/75).  7. 16 [1.3] vs 20.3 [1.7], *P=*.03  8a. 8.8 vs 16.5, NS  8b. 11.8 vs 25.9, *P=*.008  8c. 13.4 vs 18.0, NS  8d. 17.8 vs 18.5, NS  8e. 11.0 vs 11.2, NS  8f. 14.8 vs 25.6, NS  8g. 12.6 vs 8.5, NS  8h. 12.6 vs 9.7, NS  8i. 6.0 vs 6.0 (Length of stay available for only 1 of 2 patients in control group), NS  8j. 10.0 vs 18.0, NS  8k. 6.5 vs 14.0, NS  8l. 32.0 vs (0 patients), NS  8m. (0 patients) vs 30.0, NS  8n. (0 patients) vs 4.0, NS  8o. 13.0 vs (0 patients), NS  8p. 13.0 (6.9) vs 17.6 (1.6), *P=*.01  9. 7.3 [6.4] vs 8.3 [0.5], *P=*.09 | 0 | 0 |
| Begg, 1989 [11] | **N=22 vs 23 patients analyzed. 1. Number of patients achieving both peak (6-10 mg/L) and trough (1-2 mg/L) aminoglycoside levels at day 2 (main outcome).  2. Number of patients achieving both peak and trough aminoglycoside levels at day 5 (main outcome).  3. Number of patients achieving peak aminoglycoside levels (mg/L) in specific ranges at day 2.**  3a. > 10 (not prespecified)  **3b. 6–10 (main outcome)**  3c. 4-6 (not prespecified)  3d. < 4 (not prespecified)  **4. Number of patients achieving peak aminoglycoside levels (mg/L) in specific ranges at day 5.**  4a. > 10 (not prespecified)  **4b. 6–10 (main outcome)**  4c. 4-6 (not prespecified)  4d. < 4 (not prespecified)  **5. Number of patients achieving trough (mg/L) aminoglycoside levels in specific ranges at day 2.**  5a. 2-4 (not prespecified)  **5b. 1-2 (main outcome)**  5c. 0.5 – 1 (not prespecified)  5d. < 0.5 (not prespecified)  **6. Number of patients achieving trough aminoglycoside levels (mg/L) in specific ranges at day 5.**  6a. 2-4 (not prespecified)  **6b. 1-2 (main outcome)**  6c. 0.5 – 1 (not prespecified)  6d. < 0.5 (not prespecified)  Other prespecified outcomes. 7. Mean [SE] peak aminoglycoside level at day 2 (mg/L).  8. Mean [SE] trough aminoglycoside level at day 2 (mg/L).  9. Mean [SE] peak aminoglycoside level at day 5 (mg/L).  10. Mean [SE] trough aminoglycoside level at day 5 (mg/L).  11. Mean [SE] daily aminoglycoside dose (mg) during treatment.  12. Number of patients with dose changes (follow-up period NR). | 1. 6 vs 0, *P=*.007  2. NS  3a. 0 vs 0, *P*=NR  3b. 9 vs 2, *P=*.01  3c. 7 vs 7, *P*=NR  3d. 0 vs 8, *P*=NR  4a. 1 vs 0, *P*=NR  4b. 5 vs 4, NS  4c. 4 vs 8, *P*=NR  4d. 0 vs 6, *P*=NR  5a. 2 vs 3, *P*=NR  5b. 9 vs 2, *P=*.013  5c. 5 vs 6, *P*=NR  5d. 0 vs 5, *P*=NR  6a. 4 vs 2, *P*=NR  6b. 4 vs 2, NS  6c. 2 vs 6, *P*=NR  6d. 0 vs 2, *P=NR*  7. 6.49 [0.39] vs 4.27 [0.52], *P=*.001  8. 1.44 [0.22] vs 0.94 [0.21], *P=*.054  9. 7.23 [0.79] vs 5.03 [0.46],*P=*.01  10. 1.76 [0.28] vs 1.07 [0.15], *P=*.013  11. 312 [17] vs 203 [13], *P=*.001  12. *P=*.15 (14 vs 9 had no dose change; 0 vs 4 had >3 changes). | **Prespecified 1. Number of deaths (follow-up period NR).  2. Change in creatinine clearance during therapy.** | 1. 1 vs 5, *P=*.2  2. *P=*.32 (9 vs 7 patients no change; 9 vs 6 patients small reversible decreases; rest had small increases) | + | 0 |
| Hickling, 1989 [13] | **Prespecified 1. Number, proportion, of patients outside of therapeutic range (6-10 mg/L for peak and <2 mg/L for trough) at 48-72hours (and who required dose change). 2. Mean [SE] peak plasma aminoglycoside levels at 48-72 hours (mg/L). 3. Mean [SE] trough levels at 48-72 hours (mg/L). 4. Number, proportion, of patients with 48-72 hours peak plasma levels: >6 mg/L.** | 1. 5/13, 38% vs 11/14, 78% *P<*.001  2. 7.45 [0.4] vs 5.14 [0.36], *P<.*001  3. 1.58 [0.27] vs 0.87 [0.155], *P=*.02  4. 12/13, 92% vs 3/14, 21%, *P<*.001 | **Prespecified 1. Mean increase in estimated creatinine clearance during recovery.** Not specified 2. Number, proportion, of patients with increase in creatinine clearance at end of treatment. | 1. 17.5% vs 20.5%, NS  2. 7/13, 54% vs 9/14, 64%, NS;  Of 13 in intervention group:  1 = no change, 1 = 7% decrease, 3 = 25-50% decrease, 1 unaccounted for.  Of 14 in control group:  1 = no change, 4 = 0-25% decrease | + | 0 |
| **Digoxin Dosing / Monitoring** | | | | | | |
| White, 1984 [7] | **Prespecified 1. Number of physician actions related to alerts at 3 months; ratio for alert/nonalert group weighted by number of alerts days (ratio >1 indicates benefit for CCDSS group). 1a. Any action.**  1b. Serum digoxin determination ordered.  1c. Digoxin withheld.  1d. Digoxin discontinued.  1e. Digoxin dose reduced.  1f. Quinidine changed.  1g. Beta-blocking agent changed.  1h. Potassium supplement ordered.  1i. Serum potassium determination ordered.  1j. Electrocardiogram ordered | 1a. 175 vs 136; 1.22, *P<*.003  1b. 48 vs 17; 2.67, *P<*.001  1c. 27 vs 9; 2.84, *P<*.002  1d. 5 vs 2; 2.37, *P<*.14  1e. 5 vs 1; 4.73, *P<*.06  1f. 2 vs 1; 1.89, *P<*.30  1g. 4 vs 0; NR, *P<*.03  1h. 69 vs 48; 1.33, *P<*.04  1i. 117 vs 89; 1.24, *P<*.02  1j. 36 vs 29; 1.17, *P<*.25 | … | … | + | … |
| Peck, 1973 [4] | **Results unclear but prespecified at mean 3.4 weeks. 1. Mean (SD) prediction error (measured minus predicted serum digoxin level). 2. Mean between-group difference in absolute prediction error for serum digoxin level.** | 1. -0.12 (0.53), *P<*.05 vs -0.03 (0.63), NS. P-values for comparison of CCDSS vs control outcomes, NR  2. 0.06 ng/mL greater error in control group, *P<*.025 | **Prespecified at mean 3.4 weeks; N rand: 21 vs 21 1. Digoxin toxicity (12-lead electrocardiogram-assessed).  2. Congestive heart failure index.** | 1. No digoxin-related toxicity detected.  2. No between-group differences in mean changes. [Note: authors only report data for all patients as a single group.] | + | 0 |
| **Lidocaine Dosing** | | | | | | |
| Rodman, 1984 [6] | **Main outcome: plasma llidocaine levels in middle of therapeutic range (1.5 to 5.0 μg/mL).**  **1. Mean plasma lidocaine level (μg/mL) at intervals after initiation of therapy:**  **1a. 0 to 30 minutes**  1ai. 0 to10 minutes  1aii 11-30 minutes  **1b. 31 to 60 minutes**  **1c. 61 to 120 minutes**  **1d. 4 to 8 hours**  Not prespecified  2. Mean [SE] overall lidocaine infusion rate (mcg/kg/min) 3. Mean [SE] final infusion rate (mcg/kg/min) 4. Mean [SE] first-hour infusion rate (mcg/kg/min) 5. Number, proportion, of patients requiring upward adjustment of lidocaine to control arrhythmia in the first six hours of therapy. | 1a. 2.34 vs 1.44, *P<*.02  1ai. *P<*.3  1aii. *P<*.01  1b. 3.2 vs 1.60, *P<*.01  1c. 3.7 vs 2.1, *P<*.01  1d. 4.5 vs 3.0, *P<*.01  2. 39.68 [7.03] vs 35.63 [4.22], NS  3. 29.24 [5.31] vs 31.24 [2.29], NS  4. 82.68 [6.05] vs 42.27 [3.86], *P<*.01  5. 4/11, 36% vs 1/9, 11%, NS | *No outcomes were specifically prespecified; N rand = 9 vs 11  **1. Number of patients with a toxic response requiring lidocaine discontinuation or dosage reduction.** | 1. 0 vs 0 | + | 0 |
| **Miscellaneous** | | | | | | |
| Matheny, 2008 [34] | **Primary**  **1. Proportion of appropriate laboratory tests within 14 days of the clinical encounter (Medication–lab reminder): number of visits with overdue tests ordered/number of visits with overdue tests (%); adjusted odds ratio (95% CI).**  **1a. Non-steroidal anti-inflammatory drugs – Creatinine (8487 vs 9307 visits).**  **1b. Angiotensin-II receptor blockers – Creatinine (751 vs 832 visits). 1c. Metformin – Creatinine (856 vs 781 visits)**  **1d. Potassium supplement – Potassium (579 vs 751 visits).**  **1e. Potassium sparing diuretic – Potassium (761 vs 875 visits). 1f. Thiazide diuretic – Potassium (1997 vs 2508 visits). 1g. Angiotensin converting enzyme inhibitors – Potassium (2279 vs 2790 visits). 1h. Statin – Alanine aminotransferase (9441 vs 10935 visits). 1i. Thyroxine – Thyroid-stimulating hormone (897 vs 1233 visits). 1j. Therapeutic levels of carbamazapine, cyclosporine, Phenobarbital, phenytoin, Proc-NAPA, valproate (514 vs 755 visits).** | 1a. 150/442, 33.9% vs 136/428, 31.8%; 1.24 (0.71 to 2.15), *P=*.46 1b. 17/31, 54.8% vs 17/27, 63.0%; 0.24 (0.04 to 1.34), *P=*.10 1c. 7/20, 35.0% vs 6/16, 37.5%; 0.53 (0.05 to 5.34), *P=*.59 1d. 7/12, 58.3% vs 5/9, 55.5%; 0.91 (0.03 to 24.44), *P=*.96 1e. 13/19, 68.4% vs 17/28, 60.7%; 0.82 (0.12 to 5.60), *P=*.84 1f. 40/62, 64.5% vs 46/89, 51.7%; 1.30 (0.63 to 2.67), *P=*.47 1g. 57/119, 47.9% vs 40/80, 50.0%; 1.00 (0.43 to 2.30), *P=*.99 1h. 291/613, 47.5% vs 358/674, 53.1%; 0.89 (0.43 to 1.81), *P=*.74 1i. 22/38, 57.9% vs 25/44, 56.8%; 1.19 (0.40 to 3.53), *P=*.75 1j. 2/16, 12.5% vs 4/26, 15.4%; 0.55 (0.03 to 8.94), *P=*.68 | … | … | 0 | … |
| Judge, 2006 [32] | **1. Alerts followed by appropriate prescriber action during 1 year study: number (proportion); relative risk (95% CI) (prespecified).**  2. Alerts, within each category, followed-up by prescribers during 1 year study: number (proportion); relative risk (95% CI) (prespecified).  2a. Central nervous system side effects.  2b. Constipation side effects.  2c. Related to orders for warfarin.  2d. Hypokalemia.  2e. Dose recommendations.  2f. Hyperkalemia.  2g. Anticholinergic side effects.  2h. Related to orders for multiple antiplatelets.  2i. Drug interactions. 2j. Orders for phenytoin. | 1. 606/1982, 31% vs 513/1861, 28%; 1.1 (1.00 to 1.2)  2a. 78/447, 17% vs 53/427, 12%; 1.4 (1.0 to 1.9) 2b. 60/271, 22% vs 75/307, 24%; 0.91 (0.67 to 1.2) 2c. 61/248, 25% vs 19/269, 7%; 3.5 (2.1 to 5.7) 2d. 151/233, 65% vs 118/178, 66%; 0.98 (0.85 to 1.1) 2e. 20/189, 11% vs 17/206, 8%; 1.3 (0.69 to 2.4) 2f. 53/140, 38% vs 59/129, 46%; 0.83 (0.62 to 1.1) 2g. 18/75, 24% vs 13/53, 25%; 0.98 (0.53 to 1.8) 2h. 7/42, 17% vs 9/27, 33%; 0.50 (0.21 to 1.2) 2i. 10/42, 24% vs 4/30, 13%; 1.8 (0.62 to 5.2) 2j. 2/7, 29% vs 13/14, 93%; 0.31 (0.09 to 1.0) | … | … | 0 | … |
| Overhage, 1997 [21] | **Prespecified unless otherwise indicated: 1. % corollary orders with immediate compliance.  1a. Overall.**  1b. Excluding saline lock orders (not prespecified).  1c. At 1^st^ order suggestion (not prespecified).  **2. % corollary orders with compliance within 24 hours.**  **2a. Overall** 2b. Excluding saline lock orders (not prespecified).  **3. % corollary orders with compliance during hospital stay.**  **3a. Overall.**  3b. Excluding saline lock orders (not prespecified).  **4. Number of times pharmacists intervened with physicians for significant errors over 6 months.**   Not prespecified  5. Compliance with corollary orders within 24 hours for the following 25 most common triggering orders: total number of orders; % compliance (% increase).  5a. Heparin infusion.  5b. IV fluid orders.  5c. Cimetidine po.  5d. Type and cross.  5e. Insulin lente humulin.  5f. Furosemide po.  5g. Ferrous sulfate.  5h. Furosemide IV.  5i. Warfarin.  5j. Ventilator settings.  5k. Insulin NPH humulin.  5l. Vancomycin IV.  5m. Sustained release theophyllin.  5n. Gentamicin IV.  5o. Insulin reg humulin.  5p. Digoxin po.  5q. Glyburide po.  5r. Meperidine intramuscular/IV.  5s. Captopril po.  5t. Enteral feeding.  5u. Enalapril po.  5v. Kayexalate suspension.  5w. Timentin IV.  5x. Spironolactone po.  5y. Glipizide po.  6. Compliance with the following 25 most common corollary orders within 24 hours: total number of orders; % compliance (% increase).  6a. Serum creatinine.  6b. Saline lock.  6c. Serum electrolytes.  6d. Glycosylated haemoglobin A1.  6e. Activated partial thromboplastin time  6f. Serum glutamic-pyruvic transaminase (Alanine transaminase).  6g. Sodium docusate.  6h. Serum glutamic-oxaloacetic transaminase (Aspartate aminotransferase).  6i. Capillary glucose.  6j. Blood cell profile.  6k. Stool occult blood test.  6l. Prothrombin time.  6m. Theophylline level.  6n. Diphenhydramine.  6o. Platelet count.  6p. Acetominophen.  6q. Reticulocyte count.  6r. Naso-gastric feeding tube.  6s. Fe (iron)-Total iron blinding capacity.  6t. Vancomycin.  6u. Phenytoin level.  6v. Portable AP chest X-ray.  6w. Arterial and venous blood gas.  6x. Simplate bleed time.  6y. Gentamicin level. | 1a. 46.3% vs 21.9%, *P<*.001  1b. 46.4% vs 27.6%, *P<*.001  1c. 48% vs 23%, *P<*.001  2a. 50.4% vs 29.0%, *P<*.001  2b. 50.9% vs 35.3%, *P<*.001  3a. 55.9% vs 37.1%,*P<*.001  3b. 56.0% vs 43.5%, *P<*.001  4. 105 vs 156, *P=*.003  5a. 1476; 77.42% vs 40.24% (37.18%)  5b. 1061; 64.66% vs 0% (64.66%)  5c. 1055; 12.66% vs 5.18% (7.48%)  5d. 542; 22.90% vs 14.64% (8.26%)  5e. 518; 40.00% vs 31.01% (8.99%)  5f. 410; 75.38% vs 62.09% (13.29%)  5g. 394; 21.43% vs 16.47% (4.96%)  5h. 360; 60.88% vs 51.85% (-0.98%)  5i. 303; 68.18% vs 35.09% (33.09%)  5j. 242; 80.14% vs 21.78% (58.36%)  5k. 241; 52.17% vs 26.19% (25.98%)  5l. 224; 60.44% vs 44.36% (16.08%)  5m. 215; 73.33% vs 45.46% (27.88%)  5n. 197; 78.35% vs 61.00% (17.35%)  5o. 197; 53.33% vs 35.87% (17.46%)  5p. 178; 96.88% vs 84.15% (12.73%)  5q. 177; 51.28% vs 43.43% (7.85%)  5r. 177; 24.24% vs 5.41% (18.84%)  5s. 177; 74.42% vs 55.06% (19.36%)  5t. 170; 23.08% vs 7.60% (15.48%)  5u. 161; 73.68% vs 70.59% (3.10%)  5v. 161; 26.09% vs 18.48% (7.61%) Article reports difference % as 18.48 (repeat of control group %) – revised to 7.61% - could not confirm with author (no response).  5w. 161; 45.24% s 14.29% (30.95%)  5x. 158; 42.25% vs 20.69% (21.56%)  5y. 147; 47.22% vs 36.00% (11.22%)  6a. 1209; 48.28% vs 41.18% (7.10%)  6b. 1065; 64.73% vs 0% (64.73%)  6c. 1034; 87.03% vs 70.86% (16.18%)  6d. 821; 23.71% vs 7.39% (16.32%)  6e. 615; 89.21% vs 59.56% (29.65%)  6f. 569; 12.63% vs 1.87% (10.76%)  6g. 506; 79.35% vs 79.26% (0.09%)  6h. 467; 7.14% vs 0% (7.14%)  6i. 446; 30.77% vs 4.41% (26.36%)  6j. 382; 80.46% vs 51.44% (29.02%)  6k. 374; 60.94% vs 12.09% (48.85%)  6l. 320; 64.57% vs 45.52% (19.05%)  6m. 270; 75.89% vs 46.51% (29.38%)  6n. 267; 16.41% vs 7.19% (9.21%)  6o. 236; 70% vs 15.09% (54.91%)  6p. 232; 19.66% vs 14.78% (4.88%)  6q. 205; 19.66% vs 11.36% (8.29%)  6r. 170; 23.08% vs 7.60% (15.48%)  6s. 149; 12.64% vs 0% (12.64%)  6t. 143; 90.74% vs 65.17% (25.57%)  6u. 140; 73.13% vs 38.36% (34.78%)  6v. 127; 81.69% vs 33.93% (47.76%)  6w. 123; 72.60% vs 0% (72.60%)  6x. 123; 26.23% vs 0% (26.23%)  6y. 118; 90% vs 75.86% (14.14%) | **Not clearly prespecified**  **1. Mean hospital length of stay (days).**  **2. Maximum serum creatinine level during hospital stay (units not reported).** | 1. 7.62 vs 8.12 (difference -0.5, 95% CI -0.17 to 1.19), *P=*.94  2. 1.51 (1.25) vs 1.42 (0.88), *P=*.28 | + | 0 |

Abbreviations: CCDSS, computerized clinical decision support system; CI, confidence interval; GP, general practitioner; INR, international normalised ratio; IV, intravenous; NR, not reported; NS, not significant; po, per os (by mouth); PR, prothrombin ratio; PT, prothrombin time; SD, standard deviation; SE, standard error.

^a^Ellipses (…) indicate item was not assessed or could not be evaluated. Outcomes in bold font were assessed for effect.

^b^Outcomes are evaluated for effect as positive (+) or negative (−) for CCDSS, or no effect (0), based on the following hierarchy, with an effect defined as ≥ 50% of relevant outcomes showing a statistically significant difference (2*P*<.05):

- If a single primary outcome is reported, *in which all components are applicable*, this is the only outcome evaluated. (see Methods section of manuscript for definition of primary outcome).
- If > 1 primary outcome is reported, the ≥ 50% rule applies and only the primary outcomes are evaluated.
- If no primary outcomes are reported (or only some of the primary outcome components are relevant) but overall analyses are provided, the overall analyses are evaluated as primary outcomes. Subgroup analyses are not considered.
- If no primary outcomes or overall analyses are reported, or only some components of the primary outcome are relevant for the application, any reported prespecified outcomes are evaluated.
- If no clearly prespecified outcomes are reported, any available outcomes are considered.
- If statistical comparisons are not reported, ‘effect’ is designated as not evaluated (…).
