# Supplementary material for: Computerized clinical decision support systems for therapeutic drug monitoring and dosing: A decision-maker-researcher partnership systematic review
Source: Implement Sci. 2011 Aug 3;6:90. doi: 10.1186/1748-5908-6-90 (PMC3170236; doi:10.1186/1748-5908-6-90)
Supplement: Additional file 5 — Costs and CCDSS process-related outcomes for trials of therapeutic drug monitoring and dosing. Cost and CCDSS process-related outcomes for the included studies. [file 1748-5908-6-90-S5.DOCX]

**Additional file 5, Table S5. Costs and CCDSS process-related outcomes for trials of therapeutic drug monitoring and dosing^a^**

| **Study** | **CCDSS adverse effects** | **Costs^b^** | **Group comparison for CCDSS workflow** | **Practitioner satisfaction with CCDSS system** |
| --- | --- | --- | --- | --- |
| **Warfarin and Oral Anticoagulant Dosing** | | | | |
| Poller, 2008 [35-37] | Physicians in CCDSS group changed the CCDSS recommended dose on 10.9% of occasions for clinical reasons such as patient undergoing intervention, patient not following treatment, or patient had experienced a lifestyle change. |  |  |  |
| Claes, 2005 [27, 28] | ... | … | ... | Author reports that all interventions were described as useful in daily practice. CoaguChek scored highest on the question regarding the usefulness and value and was the intervention users most wished to implement. Feedback was considered the least time-consuming of the interventions. |
| Mitra, 2005 [29] | ... | Improved efficacy with CCDSS didn't come at the expense of overdosing and CCDSS did not require more frequent blood draws, an important factor that affects cost control and patient comfort. | ... | ... |
| Manotti, 2001 [27] | Experienced physicians rejected the computer suggestions <20% of the time. |  |  |  |
| Fitzmaurice, 2000 [25] | ... | Intervention cost mean £169 ($270) per patient per year; £69 ($110) per patient per year for controls. Expenses largely due to costs of setting up practice-based clinics and increased frequency of testing found within the practices. Practice-based costs significantly affected (*P*<.001) by practice size, with larger practices sustaining lower mean costs. | ... | ... |
| Ageno, 1998 [23] | Proportion of interventions manually overridden, 4.9% (3.0% when INR was >5, 1.9% when INR <5). | ... | ... | ... |
| Fitzmaurice, 1996 [20] | ... | Note: costs appear to be based on non-randomized practice. Cost per visit at the review frequency was £10.05. £45 were saved for each out-patient visit avoided, so savings were £34.95 per visit. Outlay of £2000 for software would be recouped after 92 patient visits. | ... | ... |
| **Aminophylline and Theophylline Dosing** | | | | |
| Tierney, 2005 [34] | ... | Physician intervention vs pharmacist intervention vs both interventions vs control 1. Mean (SD) direct health care charges over 12 months (US $). All NS unless noted otherwise. a. Outpatient charges. 3,142 (3,381) vs 2,814 (3,282) vs 3,177 (3,558) vs 3,129 (2,921) b. Inpatient charges. 4,864 (17,257) vs 2,519 (7,267) vs 2,475 (8,699) vs 2,671 (6,805) c. Total health care charges. 8,006 (18,720) vs 5,333 (9,400) vs 5,652 (10,579) vs 5,800 (8,536), *P*<.05 for increase with physician intervention. | ... | ... |
| **Insulin Dosing and Glucose / Glycaemic Regulation** | | | | |
| Rood, 2005 [30] | ... | ... | ... | Author comment: Not described but yes [a majority of practitioners using the CCDSS were satisfied with the system]. |
| McDonald, 1976 [5] | There was a problem with the recommendations for renal treatment, such that in some cases the computer suggestion unwarranted reductions in medication. Practitioners did not act on the erroneous recommendations. | ... | ... | ... |
| **Insulin Dosing and Glucose / Glycaemic Regulation** | | | | |
| Cavalcanti, 2009 [39] | ... | ... | 38.4% of the nurses reported that the Leuven protocol was difficult or very difficult; 13.3% reported that conventional treatment was difficult or very difficult and 11.7 % found CAIP difficult or very difficult (*P*=.78 for CAIP vs conventional treatment; *P*<.001 for CAIP vs Leuven) | From the questionnaire: 56% of the nurses would like the CAIP to be adopted as the standard protocol in their intensive car unit, 22% preferred the Leuven protocol, 15% preferred the conventional protocol, and 7% believe all the protocols were alike. |
| **Aminoglycoside Dosing** | | | | |
| Burton, 1991 [14] | ... | Costs (US $) 1. Per-patient cost avoidance (based on average bed cost and average length of stay). $1,311.45. 2. Potential benefit/cost ratio (based on 6% discount & pharmacokinetic dosing service cost of $297.23/patient). 4.09:1.00 | ... | ... |
| **Miscellaneous** | | | | |
| Overhage, 1997 [21] | ... | Mean hospital charges for intervention vs control: $8,073.52 vs $8,589.47 (difference -$515.95, 95% CI -828.41 to 1,316.85, *P*=.68). | ... | ... |

Abbreviations: CAIP, computer-assisted insulin protocol; CCDSS, computerized clinical decision support system; CI, confidence interval; INR, international normalised ratio; NS, not significant; SD, standard deviation.

^a^Ellipses (…) indicate outcome was not assessed.

^b^Costs include workflow measures such as time to process alerts if these are not directly compared between groups.
